# Intracranial Haemorrhage and Haemorrhagic Stroke in Patients with Atrial Fibrillation on Edoxaban in ETNA-AF-Europe

**DOI:** 10.3390/jcm15218575

**Published:** 2026-11-03

**Authors:** Doralisa Morrone, Richa Chhabra, Eva-Maria Fronk, Jose Souza, Paulus Kirchhof, Raffaele De Caterina, on behalf of the ETNA-AF-Europe Investigators

**Affiliations:** 1 Cardiovascular Division, Pisa University Hospital, 56124 Pisa, Italy; doralisamorrone@gmail.com; 2 Daiichi Sankyo Europe GmbH, 81379 Munich, Germany; richa.niper@gmail.com (R.C.); eva-maria.fronk@daiichisankyo.com (E.-M.F.); zecantonio@yahoo.com (J.S.); 3 Institute of Cardiovascular Sciences, University of Birmingham, Birmingham B15 2TT, UK; p.kirchhof@uke.de

**Keywords:** atrial fibrillation, edoxaban, haemorrhagic, risk factors, stroke, treatment outcomes

## Abstract

**Background/Objectives**: ETNA-AF-Europe was a post-authorization, prospective, non-interventional study evaluating edoxaban in atrial fibrillation (AF). Here, we assessed annualized rates of intracranial haemorrhage, haemorrhagic stroke, and ischaemic stroke in subpopulations of edoxaban-treated patients with AF. **Methods**: We assessed overall annualized adjudicated event rates over 4 years, including subpopulations stratified by age, renal/hepatic impairment, chronic concomitant anti-platelet use, and CHA_2_DS_2_-VASc score. **Results**: Overall, 13,164 patients were included, with a median (interquartile range) age of 75.0 (68.0, 80.0) years. Overall annualized intracranial haemorrhage rates were 0.18%/year and remained ≤0.24%/year in high-risk subpopulations, with minor rate increases observed with age (<65 years: 0.07%/year, ≥65–<75 years: 0.16%/year, ≥75 years: 0.23%/year) and CHA_2_DS_2_-VASc score (0–1: 0.04%/year, 2–4: 0.19%/year, >4: 0.24%/year). Overall annualized haemorrhagic stroke rates were 0.08%/year and remained ≤0.10%/year in high-risk subpopulations, with minor rate increases observed with age (<65 years: 0.06%/year, ≥65–<75 years: 0.08%/year, ≥75 years: 0.09%/year) and CHA_2_DS_2_-VASc score (0–1: 0.00%/year, 2–4: 0.09%/year, >4: 0.10%/year). Overall annualized ischaemic stroke rates were 0.49%/year and remained ≤1.05%/year in high-risk subpopulations; however, rates increased notably with age (<65 years: 0.21%/year, ≥65–<75 years: 0.34%/year, ≥75 years: 0.68%/year) and CHA_2_DS_2_VASc score (0–1: 0.15%/year, 2–4: 0.43%/year, >4: 1.05%/year). **Conclusions**: Annualized intracranial haemorrhage, haemorrhagic stroke, and ischaemic stroke rates in ETNA-AF-Europe were low and demonstrated no meaningful increases with renal/hepatic impairment and chronic concomitant antiplatelet use. Minor rate increases observed with higher CHA_2_DS_2_-VASc scores and increasing age were much less steep for haemorrhagic stroke than for ischaemic stroke.

## 1. Introduction

Anticoagulation is key for stroke prevention in patients with atrial fibrillation (AF) who are at a fivefold increase in stroke risk [1]. However, concerns remain over the perceived risk of life-threatening bleeding events associated with anticoagulation, such as intracranial haemorrhages, which include haemorrhagic strokes—the most lethal form of stroke, and the only haemorrhagic event considered largely irreversible [2,3,4,5,6]. Haemorrhagic strokes are associated with notably higher mortality than ischaemic strokes, with fatality rates for patients experiencing an intracerebral haemorrhage—the most common type of haemorrhagic stroke—around 40% at one month, and approximately 54% at one year [5,7]. Therefore, stroke management strategies in AF must carefully consider the risk-benefit profile of available anticoagulation therapies.

The current mainstay of AF management for stroke prevention is anticoagulation with direct oral anticoagulants (DOACs) based on evidence accumulated from randomized controlled trials and corroborated by real-world evidence over the past decade, demonstrating non-inferior efficacy and a favourable safety profile in comparison with vitamin K antagonists [8,9,10,11,12,13]. In particular, lower rates of bleeding, such as intracranial haemorrhage and haemorrhagic stroke, have been observed with DOACs compared with vitamin K antagonists [14,15,16,17]. Despite the low rates of intracranial haemorrhage observed with DOAC use, the perceived bleeding risk remains a barrier to use and can lead to suboptimal dosing or withholding of anticoagulation, particularly among patients with additional risk factors, such as advanced age, prior history of stroke, chronic concomitant anti-platelet use, and renal or liver dysfunction [3,4].

Data on the long-term use of edoxaban in patients with AF included in the Edoxaban Treatment in routiNe clinical prActice for patients with non-valvular Atrial Fibrillation (ETNA-AF-Europe; trial registration number NCT02944019; https://clinicaltrials.gov/study/NCT02944019, accessed on 21 July 2026) 4-year follow-up registry have been published previously; [18] further long-term reporting of safety data from this large real-world study help to further elucidate the bleeding risk associated with edoxaban use in patients with AF and ensure the optimal use of oral anticoagulants in this patient population. The ETNA-AF design prespecified analysis for all ischaemic and bleeding events, and specifically for ischaemic and haemorrhagic stroke [19]. Here we, therefore, report the annualized rates of intracranial haemorrhage and haemorrhagic stroke also compared with ischaemic stroke and other selected ischaemic events in patients with AF treated with edoxaban during the ETNA-AF-Europe 4-year follow-up, with a focus on various high-risk subpopulations.

## 2. Materials and Methods

The study design for ETNA-AF-Europe has been published previously [19]. Briefly, ETNA-AF-Europe was a post-authorization, prospective, non-interventional study conducted in agreement with the European Medicines Agency across 776 sites from 10 European countries (Austria, Belgium, Germany, Ireland, Italy, the Netherlands, Portugal, Spain, Switzerland and the United Kingdom), which assessed the risks and benefits of edoxaban (Daiichi Sankyo) use in patients with AF [18,19]. ETNA-AF-Europe ran from 2016 to 2022, with patients followed up every 12 ± 2 months for 4 years, and it was approved by the institutional review boards and independent Ethics Committees for all participating centres in compliance with the Guidelines for Good Pharmacoepidemiological Practice [18,19]. The study was conducted in accordance with the principles outlined in the Declaration of Helsinki (1975, revised in 2013). Adult patients were eligible for inclusion if they provided written informed consent to participate, were treated with edoxaban for AF at baseline, and were not simultaneously participating in any interventional study. No explicit exclusion criteria were specified [19].

Here, we report the annualized event rates of adjudicated intracranial haemorrhage, haemorrhagic stroke and ischaemic stroke observed in 13,164 patients included in ETNA-AF-Europe (full analysis set). We also briefly report the annualized event rates of adjudicated transient ischaemic attack and systemic embolism. Patient characteristics were recorded at baseline. The events were classified as occurring on-edoxaban or off-edoxaban treatment. On-edoxaban events were defined as events that started during treatment with edoxaban or within 3 days after edoxaban was interrupted or permanently discontinued. Off-edoxaban events were defined as events that started while the patient was not on edoxaban (i.e., at least 4 days after stopping edoxaban). Previously reported study discontinuation rate was 27.3%, among whom consent withdrawal, institution transfer, lost to follow-up, death, other, and missing were reported in 6.9%, 5.9%, 30.2%, 52.2%, 4.2% and 0.5% respectively [20]. The overall annualized event rates considered all events, i.e., events occurring on edoxaban as well as events occurring off edoxaban, while for on-edoxaban annualized event rates only events that had occurred on-edoxaban were taken into account. For intracranial haemorrhage, haemorrhagic stroke, ischaemic stroke, transient ischaemic attack and systemic embolism, both types of annual event rates were analysed and stratified by the following risk factors: age (<65 years, ≥65–<75 years, ≥75 years); renal impairment (Yes/No); hepatic impairment (Yes/No); chronic concomitant anti-platelet use at baseline (Yes/No) and CHA_2_DS_2_-VASc score (low [0–1 points], moderate [2–4 points], high [>4 points]). Chronic concomitant anti-platelet use at baseline was defined as treatment ongoing at the time of inclusion and prescribed within the previous 12 months.

### 2.1. Statistical Analysis

All data were analysed descriptively, and no confirmatory testing (including no adjustment for multiple testing) was done. All results have to be interpreted in a purely explorative way. Baseline characteristics were summarized descriptively as frequencies (*n*, %) or median (interquartile range). When calculating percentages, unknown and missing values were not considered in the denominator. For this analysis, renal impairment was defined as creatinine clearance (derived by the Cockcroft–Gault formula) of ≤80 mL/min or (if measurements of serum creatinine were not available), creatinine clearance (as reported) ≤80 mL/min or (if neither measurements of serum creatinine nor reported creatinine clearance were available) as investigator-reported renal disease (excluding Stage 1 chronic kidney disease). For this analysis, hepatic impairment was considered present if the investigator reported a bilirubin value exceeding 2× the upper limit of normal and aspartate aminotransferase/alanine aminotransferase exceeding 3× the upper limit of normal. When information on the laboratory values was not available, it was also considered present if the investigator reported a “chronic hepatic disease”. Annualized rates of the events are reported as the percentage of patients with at least one event per year, %/year (95% confidence interval).

The main difference between the annual rates for events overall and the annual rates for events on-edoxaban lies in the types of events considered and the denominators used. While for the overall annualized rates the denominator uses the individual observation times, for the on-edoxaban annualized rates, the individual exposure times are taken. Concretely, the overall annualized rates were defined as 100*number of patients with at least one event within 4 years after the baseline/sum of all individual patient observation time from the reference start date until the first event, or if the patient completed/discontinued the study without experiencing an event within 4 years after baseline until date of premature study discontinuation or death, or last available follow-up date or censoring within 48 months from the reference start date, whichever occurred first (years). The on-edoxaban annualized rates were defined as 100*number of patients with at least one event on edoxaban within 4 years after baseline/sum of all edoxaban treatment periods from reference start date until the first event on edoxaban, or if the patient completed/discontinued the study without experiencing an event on edoxaban within 4 years after baseline until (last) stop date of edoxaban, premature study discontinuation or death, or last available follow-up date or censoring after 48 months from baseline data collection point, whichever occurred first (years).

Data monitoring and refining were performed to ensure consistency across entries and minimize the volume of missing data. Incomplete edoxaban start and stop dates were imputed using a conservative approach, assuming edoxaban was received in case of uncertainty. Likewise, incomplete event dates were also imputed in a conservative manner; the earliest possible date of event onset was assumed when dates were unknown.

### 2.2. Patient and Public Involvement

Patients and/or the public were not involved in the conception, design, conduct, reporting, or dissemination plans of this research.

## 3. Results

### 3.1. Baseline Characteristics

In total, 13,164 patients were included, of whom 7461 (56.7%) were men, with a median (interquartile range) age of 75.0 (68.0–80.0) years (Table 1). Patients aged ≥75 years, those with renal/hepatic impairment, chronic concomitant anti-platelet use, and high CHA_2_DS_2_-VASc scores were more likely to be frail (as perceived by the investigator) compared with their counterparts without these characteristics (Table 1). CHA_2_DS_2_-VASc scores were higher for patients with renal impairment and chronic concomitant anti-platelet use than for their counterparts without these characteristics (Table 1). In patients for whom a history of anti-platelet therapy use in the year prior to baseline was available (*n* = 2913), aspirin was the most frequently used anti-platelet therapy (*n* = 2437, 83.7%). The mean (standard deviation) duration of edoxaban exposure was 159.3 (72.87) weeks from baseline to the 4-year follow-up period.

### 3.2. Rates of Intracranial Haemorrhage

#### 3.2.1. Overall Events

**Figure 1:**
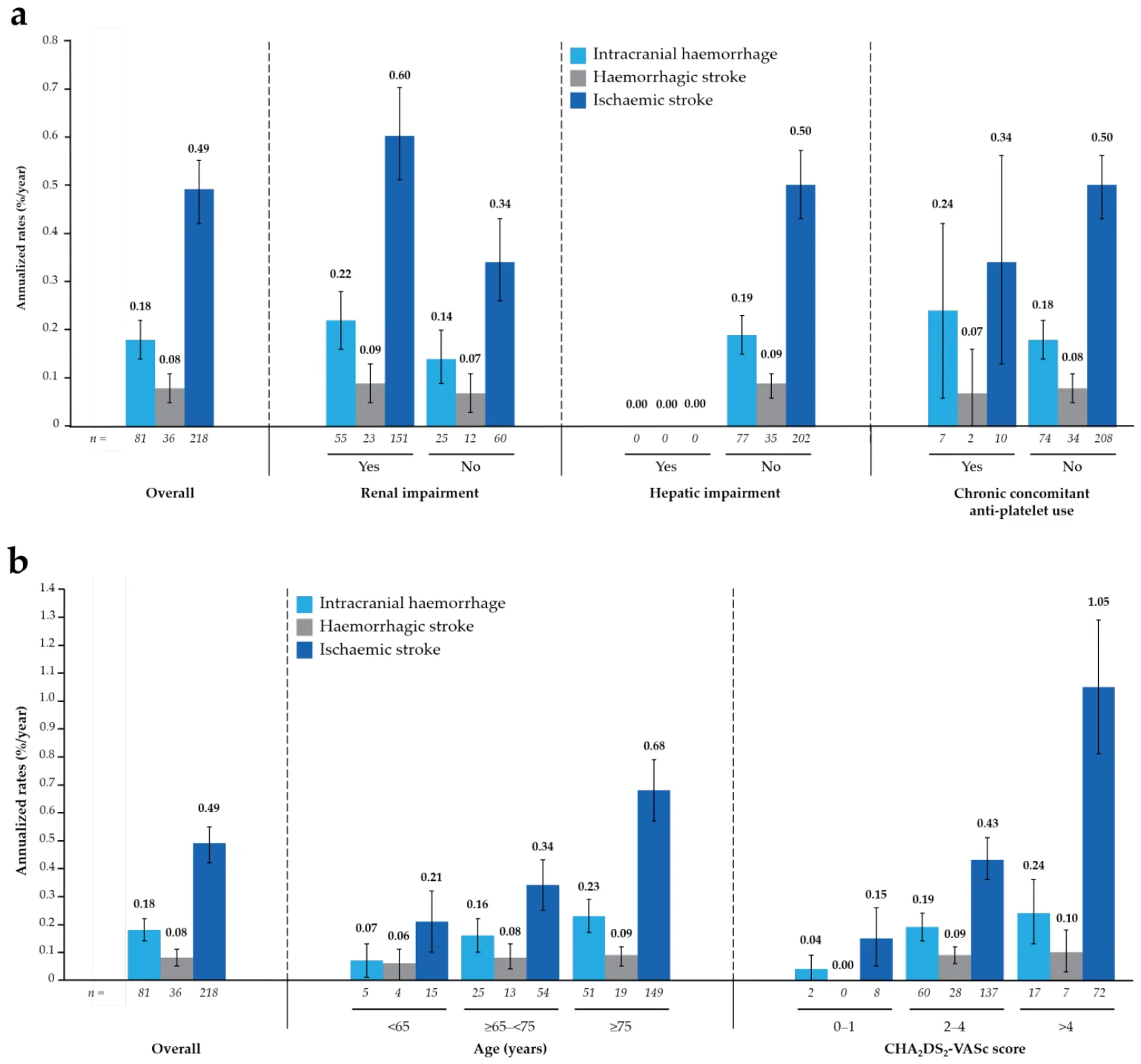
Annualized rates of intracranial haemorrhage, haemorrhagic stroke and ischaemic stroke: (**a**) by renal/hepatic impairment and chronic concomitant anti-platelet use, (**b**) by age and CHA_2_DS_2_-VASc score: overall events. Error bars represent 95% confidence intervals.

The overall annualized intracranial haemorrhage rate was 0.18%/year, with rates of 0.22%/year in patients with renal impairment and 0.14%/year in those without renal impairment. No events were reported in the small subgroup of patients with hepatic impairment (*N* = 102). Rates were slightly higher in patients with renal impairment and with chronic concomitant anti-platelet use versus patients without renal impairment and no chronic concomitant anti-platelet use, respectively (Figure 1a, Supplementary Table S1). Rates showed an upward trend with increasing age and CHA_2_DS_2_-VASc score (Figure 1b, Supplementary Table S1).

#### 3.2.2. On-Edoxaban Events

**Figure 2:**
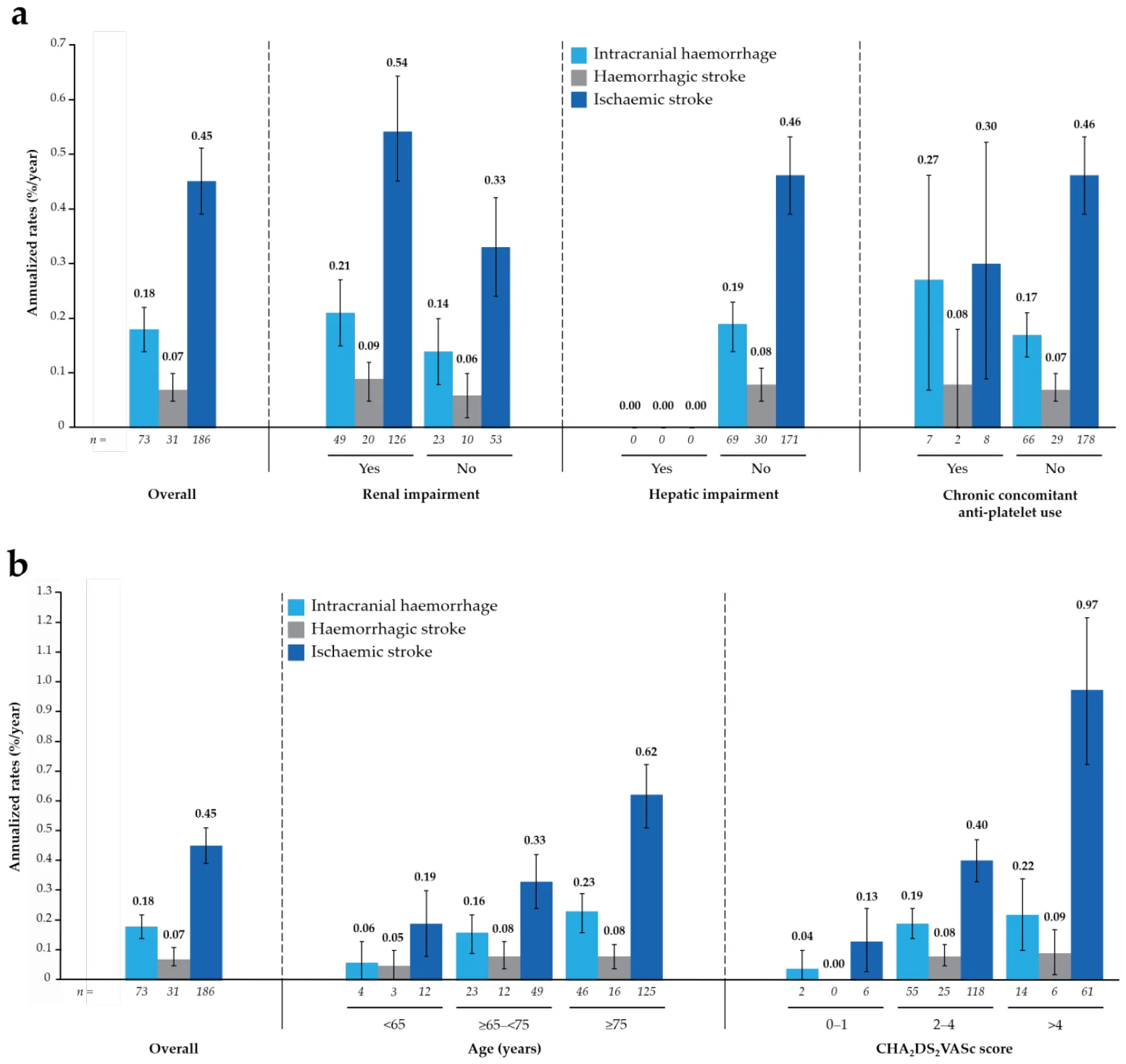
Annualized rates of intracranial haemorrhage, haemorrhagic stroke and ischaemic stroke; (**a**) by renal/hepatic impairment and chronic concomitant anti-platelet use, (**b**) by age and CHA_2_DS_2_VASc score: on-edoxaban events. Error bars represent 95% confidence intervals. On-edoxaban events were defined as events starting during treatment with edoxaban or within 3 days after edoxaban was interrupted or permanently discontinued.

The on-edoxaban annualized intracranial haemorrhage rate was 0.18%/year, with rates of 0.21%/year in patients with renal impairment and 0.14%/year in those without renal impairment. Rates were slightly higher in patients with renal impairment and with chronic concomitant anti-platelet use versus patients without renal impairment and no chronic concomitant anti-platelet use, respectively (Figure 2a, Supplementary Table S2), and not much different than what was observed under the overall events analysis. Likewise, rates showed an upward trend with increasing age and CHA_2_DS_2_-VASc score (Figure 2b, Supplementary Table S2), similar to that observed in the overall events.

### 3.3. Rates of Haemorrhagic Stroke

#### 3.3.1. Overall Events

The overall annualized haemorrhagic stroke rate was 0.08%/year, with rates of 0.09%/year in patients with renal impairment and 0.07%/year in those without renal impairment. No events were reported in the small subgroup of patients with hepatic impairment (*N* = 102). Rates were similar across renal impairment and the chronic concomitant anti-platelet use subgroups (Figure 1a, Supplementary Table S1). Rates showed a minor upward trend with increasing age and CHA_2_DS_2_-VASc score (Figure 1b, Supplementary Table S1). The overall haemorrhagic stroke rate with fatal outcome was 0.04%/year.

#### 3.3.2. On-Edoxaban Events

The on-edoxaban annualized haemorrhagic stroke rate was 0.07%/year, with rates of 0.09%/year in patients with renal impairment and 0.06%/year in those without renal impairment. Rates were slightly higher in patients with renal impairment versus patients with no renal impairment and similar across the chronic concomitant anti-platelet use subgroups (Figure 2a, Supplementary Table S2), indistinguishable from the rates observed under the overall events analysis. Rates were similar across age groups, and no incidence was observed in the 0–1 CHA_2_DS_2_-VASc score category (Figure 2b, Supplementary Table S2). The on-edoxaban haemorrhagic stroke rate with fatal outcome was 0.03%/year.

### 3.4. Rates of Ischaemic Stroke

#### 3.4.1. Overall Events

The overall annualized ischaemic stroke rate was highest among the three cerebrovascular events analysed, at 0.49%/year. The highest rate was observed in patients with renal impairment (Figure 1a, Supplementary Table S1). No events were reported in the small subgroup of patients with hepatic impairment (*N* = 102). Rates were lower in patients with chronic concomitant anti-platelet use versus those without chronic concomitant anti-platelet use (Figure 1a, Supplementary Table S1). Rates showed a noticeable increase across increasing age and CHA_2_DS_2_-VASc score categories (Figure 1b, Supplementary Table S1).

#### 3.4.2. On-Edoxaban Events

The on-edoxaban annualized ischaemic stroke rate was 0.45%/year. Rates in patients with renal impairment were higher than those without renal impairment. Rates were also higher in patients without chronic concomitant anti-platelet use versus patients with chronic concomitant anti-platelet use (Figure 2a, Supplementary Table S2). Rates showed a noticeable increase across increasing age and CHA_2_DS_2_-VASc score categories (Figure 2b, Supplementary Table S2).

### 3.5. Rates of Other Ischaemic Events

The overall annualized rates of transient ischaemic attack and systemic embolism were 0.31%/year and 0.05%/year, respectively (Supplementary Table S1). Annualized rates of transient ischaemic attack increased slightly with bleeding risk factors but remained below 1.00%/year across all analysed subgroups. Annualized rates of systemic embolism appeared to increase slightly with bleeding risk factors, other than in those aged ≥65–<75 years, and remained below 0.20%/year. For transient ischaemic attack and systemic embolism, annualized on-edoxaban event rates were similar to overall event rates (Supplementary Table S2).

## 4. Discussion

The 4-year follow-up of ETNA-AF-Europe, the largest prospective registry dedicated to a single anticoagulant, provided valuable long-term effectiveness and safety data on edoxaban use in patients with AF. In the present analysis, we report adjudicated annualized rates of intracranial haemorrhage, haemorrhagic stroke, ischaemic stroke, and selected ischaemic events across various subpopulations with bleeding risk factors. This report is unique in specifically reporting the occurrence of haemorrhagic stroke and intracranial haemorrhage, the most ominous type of bleeding, as a function of several associated variables such as renal dysfunction, concomitant use of anti-platelet medication (e.g., aspirin) and CHA_2_DS_2_Vasc score. In >13,000 edoxaban-treated patients, the overall annualized intracranial haemorrhage and haemorrhagic stroke rates remained numerically low (≤0.24%/year and ≤0.10%/year, respectively) across risk strata. This statement, in the absence of an internal comparison and in full acknowledgment of the limitations of an observational prospective registry, is supported by comparison with rates reported in the literature on age-matched AF populations with different anticoagulants [21], as well as by data on the low annualized rates of intracranial haemorrhage noted across age groups in patients receiving edoxaban in the randomized controlled trial ENGAGE AF-TIMI 48 (*N* = 21,105) [22]. These results mirror findings from a systematic review, in which the global incidence of intracranial haemorrhage over the past few decades in a non-anticoagulated population was found to be similar [23] to that reported here in the edoxaban-anticoagulated population. Additionally, no meaningful increases in the annualized rates of intracranial haemorrhage or haemorrhagic stroke were observed in patients with renal impairment, hepatic impairment, or chronic concomitant anti-platelet use.

In patients receiving anticoagulants, the risk of intracranial haemorrhage is determined by a complex interplay of factors [24]. In an analysis of intracranial haemorrhage risk in patients receiving anticoagulants enrolled in the GARFIELD-AF and ORBIT-AF registries, older age, concomitant anti-platelet use and moderate-to-severe chronic kidney disease were identified as independent predictors of intracranial haemorrhage [24]. In the present analysis, any numerical differences in intracranial haemorrhage rates across age strata were small and inconsistent. In contrast to the GARFIELD-AF and ORBIT-AF analyses, we found no meaningful increases in annualized rates of intracranial haemorrhage or haemorrhagic stroke with concomitant anti-platelet use or renal impairment. This adds to the body of evidence on the safety profile of edoxaban, which was not studied in the two registries mentioned above. Nevertheless, results should be interpreted with caution considering cross-study comparisons.

Our findings uniquely separate intracranial haemorrhages and haemorrhagic strokes from the annualized ischaemic stroke rates in this analysis, and from other bleeding events reported in the wider literature with clearly defined risk-related gradients [18,25]. The annualized rates of intracranial haemorrhage and haemorrhagic stroke remained <0.3%/year, irrespective of bleeding risk factors, somewhat contrasting the higher annualized on-treatment rates of major bleeding (0.8%/year) and major gastrointestinal (GI) bleeding (0.4%/year) reported in the full ETNA-AF-Europe 4-year analysis [18]. Here, the annualized event rates of major bleeding and major GI bleeding in the full 4-year analysis were higher in the highest (1.4%/year and 0.7%/year), intermediate (0.8%/year and 0.3%/year) in the moderate, and lowest (0.3%/year and 0.1%/year) in the lowest CHA_2_DS_2_-VASc score categories [18]. Similarly, a sub-analysis of the Italian cohort of ETNA-AF-Europe, which reported ischaemic and bleeding events in high-risk subpopulations of AF patients who received edoxaban in routine clinical care over 4 years, showed higher rates in patients with renal impairment (1.1%/year versus 0.6%/year and 0.5%/year versus 0.2%/year, respectively) [25]. The annual incidence of major bleeding was also higher in patients with chronic concomitant anti-platelet use (1.8%/year) versus those without (0.9%/year) [25]. These findings suggest that major bleeding rates increase more steeply with bleeding risk factors than intracranial haemorrhage and haemorrhagic stroke. The low annualized rates of intracranial haemorrhage and haemorrhagic stroke reported here particularly support the use of edoxaban in elderly and comorbid patients. These groups of patients are typically under-prescribed oral anticoagulants due to the perception that the risk of bleeding, and in particular for haemorrhagic stroke, which can result in irreversible damage to the brain [5,6], may outweigh anticoagulation benefits [3]. The very low risk of haemorrhagic stroke observed here favours the use of edoxaban over left atrial appendage closure, which has not shown clear superiority in patients at high bleeding risk [26,27].

### Study Strengths and Limitations

The main strength of this ETNA-AF-Europe analysis was the inclusion of a large number of patients treated with a single DOAC, with long-term follow-up data on annualized rates also for the rare events of intracranial haemorrhage and haemorrhagic stroke—particularly lethal bleeding occurrences. The prospective and non-interventional nature of the study also provided insights into intracranial haemorrhage and haemorrhagic stroke rates in an everyday clinical setting. In addition, patients were enrolled consecutively at participating centres if they were prescribed edoxaban for AF, and a central adjudication of events was enacted. The broad inclusion of over 13,000 patients from 776 sites across 10 countries, and with no exclusion criteria, enhances the generalizability of our findings.

However, this analysis was limited by the observational nature of the study, which enrolled only patients receiving edoxaban at baseline. Without a comparator arm, no direct conclusions could be drawn regarding the effectiveness or safety of edoxaban relative to other possible comparisons, and results should be interpreted with the caution of indirect comparisons. Within the ETNA-AF Europe study, only haemorrhagic stroke was consistently recorded. Subtypes of intracranial haemorrhages, such as intracerebral, subdural, subarachnoid, and distinctions between spontaneous and traumatic cases, as well as their overlap with haemorrhagic stroke, were not systematically documented. Another limitation is the rarity of the target events, increasing the risk of a Type 2 statistical error despite the very large data set and the long (4-year) follow-up period. Intrinsic to the non-interventional study design is the fact that only data provided by patients and physicians were analysed, which may have limited the accuracy of event reporting compared with event rates recorded in randomized controlled trials. Furthermore, data on patients screened but not enrolled were not systematically captured; therefore, there is the potential for selection bias if unstable or frail patients were less willing to participate in the trial. Edoxaban dose, dose-reduction criteria, inappropriate under-/over-dosing, adherence, interruptions, switching, and persistence are not reported here due to the limited scope of this manuscript. Deaths were reported as presumptive causes as annualized rates, without performing any time-to-event analysis or providing any competing risk analysis. In the ETNA-AF Europe study, renal impairment was defined broadly as CrCl ≤80 mL/min. This definition had certainly included mild age-related reduction in renal function along with relevant chronic kidney disease. The small number of patients with hepatic dysfunction is insufficient to draw clinical conclusions, and therefore the results should be interpreted with caution. Antiplatelet exposure was defined only at baseline, and changes in background therapy throughout the 4 year-long study were not captured. The use of the CHA_2_DS_2_VASc score in ETNA-AF, instead of the more recently recommended CHA_2_DS_2_VA^4^, may also constitute a limitation. We doubt, however, that the adoption of the newer, simpler score would change the main message of this study because of the minor differences between such scores. Additionally, the HAS-BLED score may be a better bleeding stratification, but has been repeatedly found to have minor discriminating ability versus CHA_2_DS_2_VASc [28]. Considering these possible limitations, confirmation of these findings in an even larger population is still needed before drawing definitive conclusions.

## 5. Conclusions

During the 4-year follow-up of the ETNA-AF-Europe study, both overall and on-edoxaban annualized event rates of adjudicated intracranial haemorrhage and haemorrhagic stroke remained consistently low, with no meaningful increase in patients with renal/hepatic impairment or concomitant anti-platelet use. Across age and CHA_2_DS_2_-VASc strata, variations in intracranial haemorrhage and haemorrhagic stroke rates were small and did not follow a clear or consistent pattern, in contrast to the progressive increase observed for ischaemic stroke noted in the same populations. In contrast with annualized ischaemic stroke rates increasing steeply with increasing risk factors, the rate increase of haemorrhagic stroke and intracranial haemorrhage was here minimal or absent. This study, therefore, provides important reassurance on the low risk of haemorrhagic stroke and intracranial bleeding among edoxaban-treated patients with atrial fibrillation enrolled in this large prospective real-world registry.

## Figures and Tables

**Table 1:** Baseline demographics and clinical characteristics.

Baseline Demographics and Clinical Characteristics, *n* (%) or Median (IQR)	Total (*n* = 13,164)	Age			Renal Impairment		Hepatic Impairment		Chronic Concomitant Anti-Platelet Use ^j^		CHA_2_DS_2_-VASc Score (Points)		
		<65 Years (*n* = 2000)	≥65 and <75 Years (*n* = 4458)	≥75 Years (*n* = 6706)	Yes (*n* = 7576)	No (*n* = 4933)	Yes (*n* = 102)	No (*n* = 11,857)	Yes (*n* = 895)	No (*n* = 12,269)	Low (0–1) (*n* = 1473)	Moderate (2–4) (*n* = 9205)	High (>4) (*n* = 2164)
Age, years	75.0 (68.0, 80.0)	59.0 (55.0, 62.0)	70.0 (68.0, 73.0)	80.0 (77.0, 84.0)	78.0 (73.0, 83.0)	68.0 (62.0, 74.0)	73.0 (67.0, 80.0)	75.0 (68.0, 80.0)	75.0 (69.0, 81.0)	75.0 (68.0, 80.0)	62.0 (56.0, 67.0)	75.0 (69.0, 80.0)	80.0 (76.0, 84.0)
Male	7461 (56.7)	1385 (69.3)	2642 (59.3)	3434 (51.2)	3751 (49.5)	3304 (67.0)	55 (53.9)	6661 (56.2)	607 (67.8)	6854 (55.9)	1357 (92.1)	5188 (56.4)	717 (33.1)
Weight, kg	80.0 (70.0, 90.0)	90.0 (78.0, 103.0)	82.0 (72.0, 94.0)	75.0 (66.0, 85.0)	74.0 (65.0, 83.0)	90.0 (80.0, 100.0)	80.5 (70.0, 97.0)	80.0 (69.0, 90.0)	80.0 (70.0, 90.0)	80.0 (70.0, 90.0)	87.0 (78.0, 100.0)	80.0 (69.0, 90.0)	75.0 (65.0, 85.0)
Frailty ^a^	1410 (11.5)	29 (1.5)	170 (4.1)	1211 (19.5)	1208 (17.0)	180 (3.9)	19 (19.2)	1349 (12.1)	143 (17.0)	1267 (11.1)	16 (1.2)	851 (9.9)	504 (24.9)
CHA_2_DS_2_-VASc score, points ^b^	3.0 (2.0, 4.0)	1.0 (1.0, 2.0)	3.0 (2.0, 3.0)	4.0 (3.0, 5.0)	4.0 (3.0, 4.0)	2.0 (2.0, 3.0)	3.0 (2.0, 4.0)	3.0 (2.0, 4.0)	4.0 (3.0, 5.0)	3.0 (2.0, 4.0)	1.0 (1.0, 1.0)	3.0 (2.0, 4.0)	5.0 (5.0, 6.0)
HAS-BLED score, points ^c^	2.0 (1.0, 2.0)	0.0 (0.0, 1.0)	2.0 (1.0, 2.0)	2.0 (2.0, 3.0)	2.0 (2.0, 3.0)	1.0 (1.0, 2.0)	3.0 (2.0, 3.0)	2.0 (1.0, 2.0)	3.0 (2.0, 3.0)	2.0 (1.0, 2.0)	1.0 (0.0, 1.0)	2.0 (1.0, 2.0)	2.0 (2.0, 3.0)
CrCl ^d^, mL/min	68.9 (52.7, 87.9)	100.3 (84.3, 118.6)	78.9 (65.0, 94.1)	57.1 (44.6, 70.6)	58.3 (46.2, 68.4)	96.4 (87.2, 110.3)	69.5 (55.1, 89.5)	68.7 (52.5, 87.8)	63.8 (49.3, 83.1)	69.3 (53.0, 88.3)	93.5 (78.5, 111.6)	68.8 (53.7, 86.7)	56.2 (42.9, 72.3)
Renal impairment ^e^	7576 (60.6)	313 (16.5)	2018 (47.7)	5245 (82.2)	–	–	–	–	–	–	326 (23.3)	5352 (61.4)	1709 (81.1)
Renal disease ^f^	3582 (29.4)	254 (13.7)	916 (22.3)	2412 (38.8)	3069 (41.7)	513 (10.6)	35 (36.5)	3404 (29.5)	293 (34.6)	3289 (29.0)	178 (13.1)	2406 (28.4)	910 (44.0)
Hepatic impairment ^g^	102 (0.9)	16 (0.9)	41 (1.0)	45 (0.7)	–	–	–	–	–	–	12 (0.9)	72 (0.9)	13 (0.6)
Chronic hepatic disease ^h^	175 (1.5)	32 (1.8)	62 (1.5)	81 (1.3)	103 (1.4)	71 (1.5)	39 (38.2)	135 (1.2)	12 (1.4)	163 (1.5)	18 (1.4)	118 (1.4)	33 (1.6)
History of anti-platelet use ^i^	2913 (22.1)	345 (17.3)	933 (20.9)	1635 (24.4)	1850 (24.4)	935 (19.0)	17 (16.7)	2645 (22.3)	895 (100.0)	2018 (16.4)	200 (13.6)	1895 (20.6)	745 (34.4)

ALT, alanine aminotransferase; AST, aspartate aminotransferase; CrCl, creatinine clearance; IQR, interquartile range; SEE, systemic embolic event; TIA, transient ischaemic attack; ULN, upper limit of normal. ^a^ Frailty was defined as perceived frailty and was based on the investigator’s own clinical binary judgement for each patient. ^b^ CHA_2_DS_2_-VASc score: heart failure (1 point), hypertension (1 point), ≥75 years old (2 points), diabetes mellitus (1 point), stroke/TIA/SEE (2 points), vascular disease (1 point), aged 65–74 years (1 point), female sex (1 point). ^c^ HAS-BLED (hypertension [1 point], CrCl ≤80 mL/min or liver disease [1 or 2 points], stroke history [1 point], prior major bleeding or predisposition to bleeding [1 point], aged >65 years [1 point], medication usage predisposing to bleeding or alcohol usage [1 or 2 points]). ^d^ Values outside of the 5–150 range were considered missing. ^e^ Renal impairment was defined as CrCl (derived by Cockcroft–Gault formula) ≤80 mL/min or (if measurements of serum creatinine were not available) CrCI (as reported) ≤80 mL/min, or (if neither measurements of serum creatinine nor reported CrCI were available) as investigator-reported renal disease (excluding Stage 1 chronic kidney disease). ^f^ Renal disease was based on the investigator’s own clinical judgement for each patient. ^g^ Hepatic impairment was considered present if the investigator-reported bilirubin value exceeded 2× ULN and AST/ALT exceeded 3× ULN. When information on the laboratory values was not available, it was also considered present if the investigator reported “chronic hepatic disease” as present. ^h^ Chronic hepatic disease was based on the investigator’s own clinical binary judgement for each patient. ^i^ History of anti-platelet use was defined as any anti-platelet treatments taken in the 12-month period prior to or at baseline. ^j^ Chronic concomitant anti-platelet use at baseline was considered present if anti-platelet treatments had been used in the past 12 months and were ongoing at baseline.

## Data Availability

De-identified individual participant data and applicable supporting clinical study documents are available on request, depending on circumstances, at https://vivli.org. In cases in which clinical study data and supporting documents are provided pursuant to the sponsor’s policies and procedures, the sponsor will continue to protect the privacy of the clinical study participants. Details on data sharing criteria and the procedure for requesting access can be found at https://vivli.org/ourmember/daiichi-sankyo/ (accessed on 21 July 2026).

## Supplementary Materials

The following supporting information can be downloaded at: https://www.mdpi.com/article/10.3390/jcm15218575/s1, Table S1: Annualized rates of cerebrovascular and other ischaemic events in the overall study population.; Table S2: Annualized rates of cerebrovascular and other ischaemic events in the on-edoxaban population; Table S3: The ETNA-AF Europe study investigator list.

## Abbreviations

The following abbreviations are used in this manuscript:

AF	Atrial fibrillation
ALT	Alanine aminotransferase
AST	Aspartate aminotransferase
CrCl	Creatinine clearance
DOAC	Direct oral anticoagulant
GI	Gastrointestinal
SEE	Systemic embolic event
TIA	Transient ischaemic attack
ULN	Upper limit of normal
