# Supplementary material for: Intracranial Haemorrhage and Haemorrhagic Stroke in Patients with Atrial Fibrillation on Edoxaban in ETNA-AF-Europe
Source: J Clin Med. 2026 Nov 3;15(21):8575. doi: 10.3390/jcm15218575 (PMC13764569; doi:10.3390/jcm15218575)
Supplement: Supplementary file 1 [file jcm-15-08575-s001.zip › jcm-4582868-supplementary.pdf]

Supplementary Table S1. Annualized rates of cerebrovascular and other ischaemic events in the overall study population.

Baseline Characteristics	Number of subjects	Annualised rate (%/year [95% CI])
Intracranial Haemorrhage		
**Overall**	81	0.18 [0.14, 0.22]
**Age (years)**		
Age <65 years	5	0.07 [0.01, 0.13]
Age >=65 and <75 years	25	0.16 [0.10, 0.22]
Age >=75 years	51	0.23 [0.17, 0.29]
**Renal impairment**		
Yes	55	0.22 [0.16, 0.28]
No	25	0.14 [0.09, 0.20]
**Hepatic impairment**		
Yes	0	NA
No	77	0.19 [0.15, 0.23]
**Chronic concomitant antiplatelet use**		
Yes	7	0.24 [0.06, 0.42]
No	74	0.18 [0.14, 0.22]
**CHA_2DS_2-VASc score**		
Low (0-1 pt)	2	0.04 [0.00, 0.09]
Moderate (2-4 pts)	60	0.19 [0.14, 0.24]
High (>4 pts)	17	0.24 [0.13, 0.36]
Haemorrhagic Stroke		
**Overall**	36	0.08 [0.05, 0.11]
**Age (years)**		
Age <65 years	4	0.06 [0.00, 0.11]
Age >=65 and <75 years	13	0.08 [0.04, 0.13]
Age >=75 years	19	0.09 [0.05, 0.12]
**Renal impairment**		
Yes	23	0.09 [0.05, 0.13]
No	12	0.07 [0.03, 0.11]
**Hepatic impairment**		
Yes	0	NA
No	35	0.09 [0.06, 0.11]
**Chronic concomitant antiplatelet use**		
Yes	2	0.07 [0.00, 0.16]
No	34	0.08 [0.05, 0.11]
**CHA_2DS_2-VASc score**		
Low (0-1 pt)	0	NA
Moderate (2-4 pts)	28	0.09 [0.06, 0.12]
High (>4 pts)	7	0.10 [0.03, 0.18]

**Ischaemic Stroke**		
**Overall**	218	0.49 [0.42, 0.55]
**Age (years)**		
Age <65 years	15	0.21 [0.10, 0.32]
Age >=65 and <75 years	54	0.34 [0.25, 0.43]
Age >=75 years	149	0.68 [0.57, 0.79]
**Renal impairment**		
Yes	151	0.60 [0.51, 0.70]
No	60	0.34 [0.26, 0.43]
**Hepatic impairment**		
Yes	0	NA
No	202	0.50 [0.43, 0.57]
**Chronic concomitant antiplatelet use**		
Yes	10	0.34 [0.13, 0.56]
No	208	0.50 [0.43, 0.56]
**CHA2DS2-VASc score**		
Low (0-1 pt)	8	0.15 [0.05, 0.26]
Moderate (2-4 pts)	137	0.43 [0.36, 0.51]
High (>4 pts)	72	1.05 [0.81, 1.29]
**Transient Ischemic Attack**		
**Overall**	140	0.31 [0.26, 0.36]
**Age (years)**		
Age <65 years	11	0.16 [0.06, 0.25]
Age >=65 and <75 years	54	0.34 [0.25, 0.43]
Age >=75 years	75	0.34 [0.26, 0.42]
**Renal impairment**		
Yes	93	0.37 [0.29, 0.44]
No	43	0.25 [0.17, 0.32]
**Hepatic impairment**		
Yes	0	NA
No	130	0.32 [0.27, 0.38]
**Chronic concomitant antiplatelet use**		
Yes	15	0.52 [0.26, 0.78]
No	125	0.30 [0.25, 0.35]
**CHA_2DS_2-VASc score**		
Low (0-1 pt)	2	0.04 [0.00, 0.09]
Moderate (2-4 pts)	93	0.29 [0.23, 0.35]
High (>4 pts)	42	0.61 [0.43, 0.80]
**Systemic Embolism**		
**Overall**	24	0.05 [0.03, 0.07]
**Age (years)**		
Age <65 years	4	0.06 [0.00, 0.11]
Age >=65 and <75 years	4	0.03 [0.00, 0.05]
Age >=75 years	16	0.07 [0.04, 0.11]

**Renal impairment**		
Yes	19	0.08 [0.04, 0.11]
No	4	0.02 [0.00, 0.05]
**Hepatic impairment**		
Yes	0	NA
No	24	0.06 [0.04, 0.08]
**Chronic concomitant antiplatelet use**		
Yes	4	0.14 [0.00, 0.27]
No	20	0.05 [0.03, 0.07]
**CHA_2DS_2-VASc score**		
Low (0-1 pt)	1	0.02 [0.00, 0.06]
Moderate (2-4 pts)	16	0.05 [0.03, 0.07]
High (>4 pts)	7	0.10 [0.03, 0.18]

Adjudicated intracranial haemorrhage, haemorrhagic stroke and ischaemic stroke events were analysed. Events that occurred within the missing category of a subgroup were not displayed. Therefore, the numbers in the displayed single categories of a subgroup did not always sum up to the number of total events. Renal impairment was defined as CrCl (derived by Cockcroft–Gault formula) ≤80 mL/min, or (if measurements of serum creatinine were not available) CrCI (as reported) ≤80 mL/min, or (if neither measurements of serum creatinine nor reported CrCI were available) investigator-reported renal disease (excluding Stage 1 chronic kidney disease). Hepatic impairment was considered present if the investigator-reported bilirubin value exceeded 2× ULN and AST/ALT exceeded 3× ULN. When information on the laboratory values was not available, it was also considered present if the investigator reported "chronic hepatic disease" as present. Chronic concomitant anti-platelet use at baseline was considered present if anti-platelet treatments had been used in the past 12 months and were ongoing at baseline. CHA2DS2-VASc score: heart failure (1 point), hypertension (1 point), ≥75 years old (2 points), diabetes mellitus (1 point), stroke/TIA/SEE (2 points), vascular disease (1 point), aged 65–74 years (1 point), female sex (1 point). Overall events were classified as occurring on-edoxaban or off-edoxaban treatment. On-edoxaban events were defined as events that started during treatment with edoxaban or within 3 days after edoxaban was interrupted or permanently discontinued. Off-edoxaban events were defined as events starting while the patient was not on edoxaban (i.e. at least 4 days after stopping edoxaban).
ALT, alanine aminotransferase; AST, aspartate aminotransferase; CrCl, creatinine clearance; SEE, systemic embolic event; TIA, transient ischaemic attack; ULN, upper limit of normal.

Supplementary Table S2: Annualized rates of cerebrovascular and other ischaemic events in the on-edoxaban population.

Baseline Characteristics	Number of subjects	Annualised rate (%/year [95% CI])
Intracranial Haemorrhage		
**Overall**	73	0.18 [0.14, 0.22]
**Age (years)**		
Age <65 years	4	0.06 [0.00, 0.13]
Age >=65 and <75 years	23	0.16 [0.09, 0.22]
Age >=75 years	46	0.23 [0.16, 0.29]
**Renal impairment**		
Yes	49	0.21 [0.15, 0.27]
No	23	0.14 [0.08, 0.20]
**Hepatic impairment**		
Yes	0	NA
No	69	0.19 [0.14, 0.23]
**Chronic concomitant antiplatelet use**		
Yes	7	0.27 [0.07, 0.46]
No	66	0.17 [0.13, 0.21]
**CHA_2DS_2-VASc score**		
Low (0-1 pt)	2	0.04 [0.00, 0.10]
Moderate (2-4 pts)	55	0.19 [0.14, 0.24]
High (>4 pts)	14	0.22 [0.10, 0.34]
Haemorrhagic Stroke		
**Overall**	31	0.07 [0.05, 0.10]
**Age (years)**		
Age <65 years	3	0.05 [0.00, 0.10]
Age >=65 and <75 years	12	0.08 [0.04, 0.13]
Age >=75 years	16	0.08 [0.04, 0.12]
**Renal impairment**		
Yes	20	0.09 [0.00, 0.10]
No	10	0.06 [0.04, 0.13]
**Hepatic impairment**		
Yes	0	NA
No	30	0.08 [0.05, 0.11]
**Chronic concomitant antiplatelet use**		
Yes	2	0.08 [0.00, 0.18]
No	29	0.07 [0.05, 0.10]
**CHA_2DS_2-VASc score**		
Low (0-1 pt)	0	NA
Moderate (2-4 pts)	25	0.08 [0.05, 0.12]
High (>4 pts)	6	0.09 [0.02, 0.17]

**Ischaemic Stroke**		
**Overall**	186	0.45 [0.39, 0.51]
**Age (years)**		
Age <65 years	12	0.19 [0.08, 0.30]
Age >=65 and <75 years	49	0.33 [0.24, 0.42]
Age >=75 years	125	0.62 [0.51, 0.72]
**Renal impairment**		
Yes	126	0.54 [0.45, 0.64]
No	53	0.33 [0.24, 0.42]
**Hepatic impairment**		
Yes	0	NA
No	171	0.46 [0.39, 0.53]
**Chronic concomitant antiplatelet use**		
Yes	8	0.30 [0.09, 0.52]
No	178	0.46 [0.39, 0.53]
**CHA2DS2-VASc score**		
Low (0-1 pt)	6	0.13 [0.03, 0.24]
Moderate (2-4 pts)	118	0.40 [0.33, 0.47]
High (>4 pts)	61	0.97 [0.72, 1.21]
**Transient Ischemic Attack**		
**Overall**	125	0.30 [0.25, 0.36]
**Age (years)**		
Age <65 years	11	0.18 [0.07, 0.28]
Age >=65 and <75 years	47	0.32 [0.23, 0.41]
Age >=75 years	67	0.33 [0.25, 0.41]
**Renal impairment**		
Yes	82	0.35 [0.28, 0.43]
No	39	0.24 [0.17, 0.32]
**Hepatic impairment**		
Yes	0	NA
No	115	0.31 [0.25, 0.37]
**Chronic concomitant antiplatelet use**		
Yes	12	0.46 [0.20, 0.72]
No	113	0.29 [0.24, 0.35]
**CHA_2DS_2-VASc score**		
Low (0-1 pt)	2	0.04 [0.00, 0.10]
Moderate (2-4 pts)	84	0.29 [0.22, 0.35]
High (>4 pts)	36	0.57 [0.38, 0.76]
**Systemic Embolism**		
**Overall**	21	0.05 [0.03, 0.07]
**Age (years)**		
Age <65 years	4	0.06 [0.00, 0.13]
Age >=65 and <75 years	4	0.03 [0.00, 0.05]
Age >=75 years	13	0.06 [0.03, 0.10]

**Renal impairment**		
Yes	16	0.07 [0.04, 0.10]
No	4	0.02 [0.00, 0.05]
**Hepatic impairment**		
Yes	0	NA
No	21	0.06 [0.03, 0.08]
**Chronic concomitant antiplatelet use**		
Yes	4	0.15 [0.00, 0.30]
No	17	0.04 [0.02, 0.06]
**CHA_2DS_2-VASc score**		
Low (0-1 pt)	1	0.02 [0.00, 0.06]
Moderate (2-4 pts)	14	0.05 [0.02, 0.07]
High (>4 pts)	6	0.09 [0.02, 0.17]

Adjudicated intracranial haemorrhage, haemorrhagic stroke and ischaemic stroke events were analysed. Events that occurred within the missing category of a subgroup were not displayed. Therefore, the numbers in the displayed single categories of a subgroup did not always sum up to the number of total events. On-edoxaban events were defined as events starting during treatment with edoxaban or within 3 days after edoxaban was interrupted or permanently discontinued. Renal impairment was defined as CrCl (derived by Cockcroft–Gault formula) ≤80 mL/min, or (if measurements of serum creatinine were not available) CrCI (as reported) ≤80 mL/min, or (if neither measurements of serum creatinine nor reported CrCI were available) investigator-reported renal disease (excluding Stage 1 chronic kidney disease). Hepatic impairment was considered present if the investigator-reported bilirubin value exceeded 2× ULN and AST/ALT exceeded 3× ULN. When information on the laboratory values was not available, it was also considered present if the investigator reported "chronic hepatic disease" as present. Chronic concomitant anti-platelet use at baseline was considered present if anti-platelet treatments had been used in the past 12 months and were ongoing at baseline. CHA2DS2-VASc score: heart failure (1 point), hypertension (1 point), ≥75 years old (2 points), diabetes mellitus (1 point), stroke/TIA/SEE (2 points), vascular disease (1 point), aged 65–74 years (1 point), female sex (1 point). Overall events were classified as occurring on-edoxaban or off-edoxaban treatment. On-edoxaban events were defined as events that started during treatment with edoxaban or within 3 days after edoxaban was interrupted or permanently discontinued. Off-edoxaban events were defined as events starting while the patient was not on edoxaban (i.e. at least 4 days after stopping edoxaban).

ALT, alanine aminotransferase; AST, aspartate aminotransferase; CrCl, creatinine clearance; SEE, systemic embolic event; TIA, transient ischaemic attack; ULN, upper limit of normal.

Supplementary Table S3: The ETNA-AF Europe study investigator list.

Collaboration Group Name	*ETNA-AF Europe investigators*	
**Collaborator First Name**	**Collaborator Surname**	**Affiliation [Department, Institution, City, Country]**
Austria		
Johann	Auer	KH St Josef Braunau, Braunau
Christian	Brenneis	Reha Zentrum Münster, Münster
Robert	Buder	Konventhospital der Barmherzigen Brüder Linz, Linz an der Donau
Hans	Domanovits	AKH - Medizinische Universität Wien, Wien
Heinz	Drexel	VIVIT Institut at the academic teaching hospital of Feldkirch, Feldkirch
Jozsef	Egresits	Klinikum Klagenfurt, Klagenfurt am Wörthersee
Alexander	Erben	Praxis Dr Höll Peter, Stainz
Hamid	Fard	Hamid Oberrat Schirasi-Fard, Wien
Johannes	Foechterle	Ordination, Linz
Michael	Gottsaumer-Wolf	Office of Michael Gottsauner-Wolf, MD, Wien
Marianne	Gwechenberger	Allgemeines Krankenhaus Medizinische Universität Wien, Wien
Karl-Heinz	Karner	Privatklinik, Villach
Peter	Kaserer	Privatklinik, Salzburg
Petra	Kleewein	Ordination, Fohnsdorf
Alexander	Kober	Ordination, Sankt Aegyd am Neuwalde
Georgios	Kollias	Krankenhaus der Elisabethinen Linz, Linz
Gerald	Maurer	Ordinationszentrum an der Wiener Privatklinik (Ordination Group Practice Center), Wien
Marcus	Müllner	Internistisches Zentrum Brigittenau, Wien
Hans	Offenbacher	LKH Knittelfeld, Knittelfeld
Ventzislav	Petkov	AKH - Medizinische Universität Wien, Wien
Paul	Pinter	Ordination Pinter, Leobersdorf
Gerhard	Puhr	KRAGES Burgenlaendische Krankenanstalten - LH Güssing, Güssing
Anna	Rab	LKH Villach, Villach
Georg	Röggla	Landesklinikum Neunkirchen, Neunkirchen
Daniel	Scherr	Universitätsklinik Graz, Graz
Stephan	Schoiswohl	LKH Steyr, Steyr
Peter	Siostrzonek	A Oe Kh Der Barmherzigen Schwestern, Linz
Reinhard	Sock	Gruppenpraxis, Wien
Clemens	Steinwender	Kepler Universitätsklinikum, Linz
Thomas	Sycha	General University Hospital of Vienna, Vienna
Milan	Vosko	Kepler Universitätsklinikum, Linz
Norbert	Watzinger	LKH Feldbach, Feldbach
Heinrich	Weber	Kardio Weberm, Wien
Thomas	Weiss	Klinik Ottakring, Wien
Andreas	Winkler	Klinik Pirawarth, Bad Pirawarth
Gudrun	Zweiker	Praxis Dr Zweiker Gudrun, Straden
Belgium		
Becker	Alzand	AZ Glorieux, Ronse
Ruben Casado	Arroyo	Cliniques Universitaires de Bruxelles Hopital Erasme, Brussels
Philip-Emmanuel	Baetslé	St-Dimpna Ziekenhuis, Geel

Ivan	Blankoff	CHU de Charleroi - Hôpital André Vésale, Montigny-le-Tilleul
Tim	Boussy	AZ Groeninge, Kortrijk
Raf	Brouns	Universitair Ziekenhuis Brussel, Brussels
Axel	Caenepeel	Practice Dr Axel Caenepeel, Willebroek
Yvan	Calozet	Le Quartier du Furgy 16, 6887 HERBEUMONT, Gribomont
Luc	Capiau	BVBA Dr Luc Capiau, Wetteren
Emmanuel	Catez	C H U Brugmann - Site Victor Horta, Brussels
Hendrik	Celen	Regional Ziekenhuis Heilig Hart, Leuven
Stéphan	Chevalier	Centre de Cardiologie Ottignies, Ottignies
Frank	Cools	A Z Klina, Brasschaat
Carlo	de Asmundis	Universitair Ziekenhuis Brussel, Brussels
Nina	De Klippel	ETC Jessa Ziekenhuis, Hasselt
Filip	De Man	Europa Ziekenhuizen, Brussels
Michel	De Pauw	Universitair Ziekenhuis Gent, Gent
Tom	De Potter	Onze Lieve Vrouw Ziekenhuis, Aalst
Marc	De Tollenaere	Algemeen Ziekenhuis West, Veurne
Roeland	Dierickx	Huisartsen Dierickx, Westouter
Karl	Dujardin	AZ Delta, Roeselare
Philippe	Evrard	Clinique CHC MontLégia, Liège
Dirk	Faes	Maria Ziekenhuis Noord-Limburg vzw, Overpelt
Marie-Dominique	Gazagnes	C H U Brugmann - Site Victor Horta, Brussels
Peter	Goethals	Clinique St Jean, Brussels
Pierre	Hausman	Grand Hôpital de Charleroi, Charleroi
Hein	Heidbuchel	Universitair Ziekenhuis Antwerpen, Edegem
Stéphane	Heijmans	Research Link, Linkebeek
Dimitri	Hemelsoet	Universitair Ziekenhuis Gent, Gent
Etienne	Hoffer	C H R de la Citadelle, Liège
Geert	Hollanders	Private Practice Cardiology, De Pinte
Geert	Huisarts	Huisartsenpraktijk Vileyn bvba, Blankenberge
Fernand	Lenoir	médecine générale, Roux
Rachid	Maamar	Centre Hospitalier Hutois, Huy
Georges	Mairesse	Cliniques du Sud-Luxembourg, Arlon
Frederic	Marenne	St-Nikolaus Hospital, Eupen
Guido	Mehuys	Grote Hulststraat, Tielt
Jaak	Mortelmans	Oostham
Gaetano	Paparella	Cliniques de l'Europe St-Michel, Brussels
Luc	Piérard	CHU de Liège
Bruno	Pirenne	Clinique Saint-Pierre, Ottignies
Frank	Provenier	AZ Maria Middelares, Gent
Peter	Put	St-Franciskus Ziekenhuis, Heusden-Zolder
Maximo	Rivero	Ziekenhuis Oost-Limburg, Genk
Raf	Roelandt	VZW Regionaal Ziekenhuis Jan Yperman, Leper
Tom	Rossenbacker	Imeldaziekenhuis, Bonheiden
Tom	Sarens	AZ Sint-Blasius, Dendermonde
Bruno	Schwagten	ZNA Middelheim, Antwerpen
Mehran	Tahmaseb	AZ Sint-Jan, Oostende

Rene	Tavernier	AZ Sint-Jan, Brugge
John	Thoeng	AZ Turnhout - Campus Sint-Elisabeth, Turnhout
Geert	Valgaeren	AZ Monica, Deurne
Peter	Vanacker	AZ Groeninge, Kortrijk
Thomas	Vanassche	UZ Leuven, Leuven
Hans Vandek	Vandekerckhove	AZ Sint-Lucas, Gent
Philippe	Vanduynhoven	Algemeen Stedelijk Ziekenhuis, Aalst
Jos	Vanhoof	Artsengroep Zorgpoort, Lommel
Geert	Vanhooren	AZ Sint-Jan, Brugge
Filip	Vanstechelman	St Elisabeth Ziekenhuis, Zonnegem
Dirk	Verleyen	AZ Sint-Lucas, Brugge
Stefan	Verstraete	AZ Zeno, Knokke-Heist
Geert	Vervoort	AZ Sint-Maarten, Mechelen
Johan	Vijgen	ETC Jessa Ziekenhuis, Hasselt
Joeri	Voet	Vitaz, Sint-Niklaas
Olivier	Xhaet	CHU UCL Namur, Mont-Godinne, Yvoir
Laetitia	Yperzeele	Universitair Ziekenhuis Antwerpen, Edegem
Germany		
Muwafeg	Abdel-Qader	Praxis Abdel-Qader, Winsen
Patrick	Abdul-Malak	Gemeinschaftspraxis Dr Patrick Abdul-Malak & Reinhard Lieb, Bahrdorf
Wolfgang	Alberecht	Praxis Dr Wolfgang Albrecht, Heusenstamm
Alexander Klein	Alexander	Kardiologische Gemeinschaftspraxis Dres Klein & Neumann, Stuttgart
Kahtan	Alkopepa	Kardiologische Praxis Nienburg, Nienburg
Andree	Alleres	Kardiologie an der Paulikirche, Braunschweig
Tamer	Altilaer	Kardiologische Gemeinschaftspraxis Gelsenkirchen, Gelsenkirchen
Ayham	Al-Zoebi	Kardiologische Praxis Dr Al-Zoebi, Wermsdorf
Yuriy	Amdur	Praxis Dr med Yuriy Amdur, Nürnberg-St Johannis
Martin	Andraessy	Fürst-Stirum-Klinik Bruchsal, Bruchsal
Karl-Friedrich	Appel	B Braun Cardio Zentrum Kassel MVZ GmbH, Kassel
Haydar	Aslane	Praxis H Aslan, Wetzlar
Christoph	Axethelm	Hausärztlich-Kardiologisches MVZ „Am Felsenkeller“ GmbH, Dresden
Markus	Baar	Kardiologische Praxis Northeim, Northeim
Klaus	Bachhueber	Praxis Dr med Bachhuber, Oberdorf
Alain	Barakaet	Gemeinschaftspraxis Alain Barakat und Helene Willems, Duisburg
Marius	Barbueia	Ärztezentrum Hammelburg/Dres Edgue/Barbuia/Linke, Hammelburg
Sylvia	Baumbeach	Gemeinschaftspraxis Dr med Sylvia Baumbach und Dr Elke Reichelt, Apolda
Anselm	Bäumeer	Praxis für Innere Medizin, Kardiologie, Pneumologie, Köln,
Karsten	Beckeer	Praxis Karsten Becker, Wesel
Bernd	Beckere	Praxis am Markt, Essen
Georg	Behnese	Praxis Georg Behnes, Karlsruhe
Fahri	Beqa	Herzpraxis Hohenschönhausen, Berlin
Ralf	Berg	Praxis im Alten Rathaus, Einzelpraxis Dr med R Berg, Ühlingen-Birkendorf
Alex-Mihael	Berinde	Praxis Dr Alex Berinde, Neu-Isenburg

Robert	Bernaet	Westpfalz-Klinikum GmbH - Standort I, Kaiserslautern
Jan	Beyer-Westendorf	Universitätsklinikum Carl Gustav Carus TU Dresden, Dresden
Annette	Biedeermann	Praxis Dr med Annette Biedermann, Blankenhain
Hans-Walter	Bindig	Gemeinschaftspraxis Dr med Hans-Walter Bindig und Carl-André Bystron, Georgensgmünd
Annette	Birkeenhagen	Praxis Dr med Annette Birkenhagen, Stollberg
Ralf	Birkemeeyer	Herzklinik Ulm, Dr Haerer und Partner, Ulm
Erwin	Blessieng	SRH Klinikum Karlsbad-Langensteinbach GmbH, Karlsbad
Michael	Bögeel	Praxen Dr Bögel, Völk und Kollegen, Nürnberg
Mirko	Böhme	Praxis Dr Mirko Böhme, Sulzberg
Andreas	Bollemann	Herzzentrum Leipzig GmbH, Leipzig
Dirk	Bonfilse	Internistisch-Kardiologische Praxis Kandel, Kandel
Harald	Borgmeann	Allgemeinmedizinisch Internistische Praxis Dr med Harald Borgmann, Radebeul
Ralph	Bosch	Cardio Centrum Ludwigsburg Bietigheim, Ludwigsburg
Philipp	Boyée	Kardiologische Gemeinschaftspraxis am Park Sanssouci, Potsdam
Cornelia	Braeuer	Praxis Dr Brauer, Hamburg
Roland	Braune	Praxis Dr med Roland Braun, Unterschneidheim
Bernadett	Breado	Praxis für Angiologie und Hämatologie -Dr med Bernadett Brado, Heidelberg-Neuenheim
Carsten	Brinekmann	Praxis für Allgemeinmedizin Dr med Carsten Brinkmann, Moormerland-Veenhusen
Karl	Bruck	Praxis Dr med Karl Bruck, Pirmasens
Markus	Buresech	Kardiologische Gemeinschaftspraxis Dr med Kösler, Dr med Bauer, Dr med Buresch und Prof Dr med Niederer, Regensburg,
Hermann	Burkeei	Praxis Dr med Hermann Burkei, Kaiserslautern
Carlo	Buttroen	Praxis Carlo Buttron, Groß Rohrheim
Marco	Campo dell' Orto	Sportklinik Bad Nauheim, Bad Nauheim
Roy Ben	Chur	Praxis für Allgemeinmedizin, Großes Palais Meiningen – Das Ärzte- und Gesundheitszentrum, Meiningen
Matthias	Claeus	Praxis Dr Matthias Claus, Berlin-Köpenick
Tillmann	Daheme	Universitätsklinikum Ulm, Ulm
Kai-Alexander	Dähmlow	Praxis Dr Kai-Alexander Dähmlow, Murrhardt
Thomas	Dengleer	SLK-Kliniken Heilbronn GmbH - Klinikum am Plattenwald, Bad Friedrichshall
Michael	Denkeinger	Agaplesion Bethesda Klinik Ulm, Ulm
Mark	Deubiel	Praxis Dr Dubiel, Hamburg
Michael	Drexeler	Cardiopraxis Mainz und Ingelheim GbR - MED Facharztzentrum, Mainz
Rainer	Drostee	Praxis Dr med Rainer Droste, Heilbronn
Mustafa	Duraek	Kardiologische Schwerpunktpraxis, Heidelberg
Michael	Dusoeld	Dres med Claudia Wankmüller u Michael Dusold, Rottweil
Hans-Juergen	Dworatzek	Praxis N Nabo/H J Dworatzek, Bad Abbach
Katrin	Eberheard	Hausärztlich-Kardiologisches MVZ „Am Felsenkeller" GmbH, Pirna
Hans-Holger	Ebert	Gemeinschaftspraxis Dres med Stenzel, Ebert & Otto, Riesa
Andreas	Eckee	Praxis Dr med Andreas Ecke, Rottweil
Egbert	Eggeres	Kreiskrankenhaus Torgau - Johann Kentmann - gGmbH, Torgau
Dirk	Eiser	Gemeinschaftspraxis Dres med Pfeffer Sauer Eiser, Lindenberg
Enno	Eißfelleer	Praxis Dr med Eißfeller, Woellstein
Volker	Eissieng	MVZ Birkenallee GmbH, Papenburg

Ralf	Engelhaerd	Internistische Gemeinschaftspraxis Dr med Engelhard/Dr med Wihl, Frankenberg
Fikret	Er	Klinikum Gütersloh gGmbH, Gütersloh
Erik	Eriek May	MVZ im Rolshover Hof AmKaRe Poll - Dres Gysan, Heinzler, May GbR, Köln,
Jan	Ernstbereger	Medizinisches Versorgungszentrum am Küchwald GmbH, Chemnitz
Markus	Faghieh	Studienzentrum Bocholderstrasse, Dres med Faghih/Zühlke, Essen
Dorothea	Falekenstein	Praxis Dr Falkenstein and Dr Löser, Neustadt
Peter	Falk	Herz-Kreislauf-Klinik Bevensen AG, Bad Bevensen
Christian	Feechtrup	Praxis für Innere Medizin, Münster
Stephanie	Fiechtner	Klinikum der Universität München, München
Sven	Fischer Sven	Harzklinikum Dorothea Christiane Erxleben GmbH, Quedlinburg
Herbert	Fissean	Praxis Dres Fissan und Werner, Bad Lippspringe
Udo	Frädriche	Praxis Dr med Udo Frädrich, Oberhausen
Peter W	Frank	Praxis Dr med Peter W Frank, Gröbenzell
Dirk	Franke	Institut für Hämostaseologie GmbH, Möser
Emanuel	Friteschka	Praxis Prof Dr med Emanuel Fritschka, Bad Brückenau
Jürgen	Fritsech	Kardiologie Fritsch - Facharztzentrum am Heilig Geist-Gesundheitszentrum, Köln-Longerich
Christoph	Gaerlichs	DIAKO Krankenhaus gGmbH, Flensburg
Andreas	Gammeel	Praxis Dr med Andreas Gammel, Mössingen
Manfred	Ganze	Praxis Dr Rolf Dietzel und Kollegen - ortsübergreifende Gemeinschaftspraxis, Dannenfels
Oliver	Gastmeann	Ilm-Kreis-Kliniken Arnstadt-Ilmenau gGmbH, Arnstadt
Johann Christoph	Geller	Zentralklinik Bad Berka GmbH, Bad Berka
Sabine	Genth-Zotz	Katholisches Klinikum Mainz, Caritas Werk St Martin Gem Träger und Betriebsf GmbH, Mainz
Conrad	Genz	Universitätsklinikum Magdeburg A ö R , Magdeburg
Uwe	Gerbauleet	Praxisgemeinschaft Dr Biesenbaum und Dr Gerbaulet, Löhne
Detlef	Germaenn	Hausarztpraxis Anna Lorenz und Detlef Germann, Wetzlar
Tibo	Gerrietes	Bürgerhospital Friedberg Neurologie, Friedberg
Jan	Gerrit Voigt	Praxis Dr med Jan-Gerrit Voigt, Dorsten
Dirk	Glatzele	Praxis Dr med Glatzel, Hannover
Andreas	Göttee	St Vincenz-Krankenhaus GmbH, Paderborn
Carsten	Grabeowski	Allgemeinarztpraxis im PueD, Dortmund
Uwe	Gremmlere	MVZ DaVita Kardiologisches Zentrum Peine GmbH, Peine
Andreas	Grevee	Innere Medizin im Gesundheitszentrum, Ahrensburg
C. Michael	Gross	Johanniter-Krankenhaus Genthin - Stendal gGmbH, Stendal
Johannes	Haaes	CIMS UG mbh, Bamberg
Günter	Häfelee	Praxis für Allgemeinmedizin Dr med Günter Häfele, Denzlingen
Andreas	Hageenow	Praxis Dr med Andreas Hagenow, Elsterwerda
Dirk	Hagemanen	Gemeinschaftspraxis Dres Hagemann, Dürfeld und Breiderhoff, Essen
Afif	Haj-Yehia	Überörtliche Berufsausübungsgemeinschaft - Dres med Beate Zygan, Thomas Reiff, Stephan Linse, Afif Haj-Yehia, Moers
Frank	Hamanne	Klinikum Konstanz, Konstanz
Detlef	Hammeerschmidt	Praxis Dr med Detlef Hammerschmidt, Bad Krozingen
Bernd	Hammere	Gemeinschaftspraxis PD Dr med Bernd Hammer und Alexander Schilling, Bechhofen
Tilo	Hanfstiengl	Gemeinschaftspraxis Hanfstingl, Erlensee

Sabine	Hanseen	Praxis Dr med Sabine Hansen, Unterwellenborn (OT Goßwitz)
Stefan	Hardte	Kardiologie im Friedrichspalais, Bruchsal
Frank	Heckmaenn	Gefäßzentrum Dr Heckmann und Kollegen, Neckargemuend
Paul	Hegemanen	Gemeinschaftspraxis Dr med Paul Hegemann/Dr med Claus Franck, Ingelheim
Joachim	Heiseters	Praxis Dr med Joachim Heisters, Kamp-Lintfort Gunter Hergdt, Praxis Dr med Gunter Hergdt, Obermichelbach,
Stefan	Hetzeel	Gemeinschaftspraxis Dres Hetzel & Grewe-Kemper, Greven
Venesa	Hild	Praxis Dr med Venesa Hild, Bad Soden-Salmünster
Jörg A	Hintze	Hausarztpraxis und Diabetologische Schwerpunktpraxis Dr med Jörg A Hintze und Dr med Martin Grundner, Hainburg
Stefan	Hochreeuther	Hassberg-Kliniken Haus Hassfurt, Hassfurt
Joachim	Hocke	Gemeinschaftspraxis Klaus Peter Dausend und Joachim Hock, Oberkirchen
Thomas	Hohenestatt	Hausarzt-Praxis - Dr Thomas Hohenstatt Dr med Antonia Dengler, Ursensollen
Andreas	Hölsecher	Gemeinschaftspraxis im Altstadt-Carree Dres Simon, Schwuchow, A u C Hölscher, I Olbert und M Conze, Fulda
Manja	Hubalde	Technische Universität Dresden, Dresden
Burkhard	Hügel	Marienhaus Klinikum Neuwied, Neuwied
Hüseyin	Incee	Universitätsmedizin Rostock, Rostock
Ulrich	Jäck	Praxis Dr med Ulrich Jäck, Balingen
Frank Stephan	Jäger	Kardiologie Gemeinschaftspraxis Kassel, Kassel
Sigmund	Jakoeb	Praxis Sigmund Jakob, Weinsberg
Dieter	Jänisch-Bernstein	Kardiologische Praxis Waldstraße, Karlsruhe
Gerhard	Janßeen	Kardiologische Gemeinschaftspraxis am Park Sanssouci, Potsdam
Moritz	Jennieches	Praxis Dr Jenniches, Bad Salzdetfurth
Kurt	Jeocham	Internistisches Facharztzentrum mit Dialyse Fachbereich Kardiologie - Angiologie, Memmingen
Reinhold	Jerwan-Keim	Gemeinschaftspraxis Dr med Reinhold Jerwan-Keim, Renate Metz, Dietzenbach
Nils	Jonas	Praxis Dr med Nils Jonas, Heitersheim
Wolfgang	Junegmair	Kardiologische Praxis Dr med Wolfgang Jungmair, Bad Homburg
Martin	Kajzaer	Gemeinschaftspraxis Vogelstang, Mannheim
Jan	Keähler	Klinikum Herford, Herford
Sebastian	Keeßler	iKardio, Hürth
Matthias	Keiem	Kardiologische Gemeinschaftspraxis RT, Reutlingen
Dag-Alexander	Keilhau	Medizinisches Versorgungszentrum für Innere Medizin, Hamburg
Stephan	Keldeenich	Praxis Dr Stephan Keldenich, Aachen
Winfried	Kesesler	Überörtliche Gemeinschaftspraxis - Dres Kessler, Kessler-Schwigon & Kollegen, Erlangen
Jörg	Keynast	Kardiologie Alsterquelle, Praxis Dr Jörg Kynast, Henstedt-Ulzburg
Holger	Killaet	Kardiologische Praxis Holger Killat, Haßloch
Dirk	Killermeann	DRK-Krankenhaus Grevesmühlen gGmbH, Grevesmühlen
Klaus Bergmann	Klaus	Internistische Schwerpunkt Praxen, Erlangen
Norbert	Kleien	Klinikum St Georg gGmbH, Leipzig
Karin	Klein	Gemeinschaftspraxis Dres Klein, Schreiber & von Petzinger-Kruthoff, Remagen
Waldemar	Kliemek	Dr Klimek MVZ GmbH, Neuburg an der Donau
Carsten	Klinegner	Universitätsklinikum Jena, Jena
Martin	Klutmeann	Praxis für Kardiologie Aachen GbR, Aachen

Grigorios	Koerosoglou	GRN-Klinik Weinheim, Weinheim
Steffen	Kolsechmann	Kardiologen am Fetscherplatz - Gemeinschaftspraxis für Kardiologie Dr med Michael Günther & Dr med Steffen Kolschmann, Dresden
Konrad Binder	Konrad	Praxis Dr med Binder, Dreieich-Sprendlingen
Andreas	Kopfe	Praxis Dr med Andreas Kopf, Berlin
Vivion	Koppaetsch	Praxis Dr med Vivion Koppatsch, Bad Buchau
Jürgen	Kraffet	Praxis Jürgen Krafft, Zirndorf
Fabian	Krämeer	Zentrum für Prävention und Rehabilitation, Praxisgemeinschaft Dr med Ulrich Overhoff, Dr med Fabian Krämer, Siegen,
Roland	Krämeer	Hausärztliche Praxisgemeinschaft Dr med Roland Krämer und Dr med Peter Rauh, Berlin-Neukölln
Daniel	Kretzeschmar	Universitätsklinikum Jena, Jena
Stephan	Kreuetz	Praxis Dr med Stephan Kreutz, Maxdorf
Manuel	Kronsechnabl	Gemeinschaftspraxis Dr med Bruno Kronschnabl, Dr med Manuel Kronschnabl, Sonja Römer-Kronschnabl, Regen,
Christian	Küehne	Kardiologische Gemeinschaftspraxis Dres Loebe, Weißbrodt, Leipzig
Thomas	Kuhl	Praxis für Kardiologie, Gesundheitsförderung und Prävention, Dormagen
Matthias	Lägeer	Praxis Dr Matthias Läger, Merseburg
Felix	Lampe	Kreiskrankenhaus Emmendingen, Emmendingen
Wasilios	Lazearidis	Innere am Stadtpark Dr med Martin Laser, Nürnberg
Jörg	Leangel	Praxis für Kardiologie Dr med Jörg Langel, Gera
Hassan	Lebbeed	Herzcarre Kardiologie Bad Homburg, Bad Homburg
Hugo	Leoeber	Internistische Praxis Hugo Loeber, Weiskirchen
Jan	Leokies	Gemeinschaftspraxis Dres Frank Eickhoff und Jan Lokies, Berlin
Sven	Linzbaceh	Kardiocentrum Frankfurt an der Klinik Rotes Kreuz, Frankfurt
Josef	Lißmanen	Praxis Dr med Josef Lißmann, Homburg
Christian	Loeges	Kariologische Gemeinschaftspraxis Mosbach Dres Haney/Gehrig/Loges, Mosbach
Thomas	Loeseeken	Praxis für Allgemeinmedizin Dr Loeseken, Rohrberg
Sabine	Lorenez	Praxis Dr med Sabine Lorenz, Bochum
Stephan	Lüdeers	St Josefs-Hospital, Cloppenburg
Norbert	Ludweig	Gemeinschaftspraxis Prof Dr med Ludwig und Dr med Honl, Willich-Anrath
Thomas	Ludwieg	Praxis Dr med Thomas Ludwig, Farchant
Carola	Lüke	Praxisgemeinschaft Jerichow, Schönhausen
Christian	Maehnkopf	Klinikum Coburg GmbH, Coburg
Matthias Schulze	Matthias	Asklepios Klinikum Schwalmstadt, Schwalmstadt
Sony	Mayerheeim	MVZ Gropiusstadt, Berlin
Eckhard	Meiseel	Praxisklinik Herzkreislauf am Wasa-Platz, Dresden
Frank	Menzele	Praxis für Kardiologie und Angiologie Dr med Frank Menzel, Dessau-Roßlau
Charlotte	Metreveli-Wiese	Praxis Drs Wiese/Metreveli-Wiese, Osnabrück
Michael	Metzee	Universitätsklinikum Leipzig AöR, Leipzig
Holger	Micheel	Gemeinschaftspraxis Michel und Leffler, Lutherstadt Eisleben
Veselin	Mitreovic	Kerckhoff-Klinik Forschungsgesellschaft mbH, Bad Nauheim
Heiner	Mülleer	Berufsausübungsgemeinschaft Dres med Kolitsch/Müller, Katzhütte
Karsten	Mülleer	Praxis Dr med Karsten Müller, Gräfenhainichen
Stefan	Mülleer	Kardiologische BAG - Dr med Glatthor, Dr med Müller, Dr med Schlotterbeck und Dr med Trompler, Weingarten,
Walter	Mülleer	Praxis für Herz-Kreislauf Erkrankungen, Flensburg

Mathis	Münchebach	Praxis Prof Staedt/Dr med M Münchbach, Speyer
Mohammed	Nateour	Heidelberger Praxisklinik für Innere Medizin, Kardiologie und Pneumologie, Heidelberg
Jens	Nettelreodt	Herz-/Gefäßpraxis Morsbach am Teufelsbrunnen, Morsbach
Peter	Nordbeeck	Universitätsklinikum Würzburg, Würzburg
Christian	Oeehlwein	Praxis Dr med Christian Oehlwein, Gera
Eva	Oelesch	Überörtliche Berufsausübungsgemeinschaft - Dres med Knöbel/Pelz-Knöbel/Olesch/von Hassel/Spaltmann, Straubing
Christian	Opeitz	DRK Kliniken Berlin Westend, Berlin
Karl Josef	Osterziel	Prof Dr med Karl Josef Osterziel und Peter Wittmann Oberpfalz GbR, Amberg
Ulrich	Overheoff	Zentrum für Prävention und Rehabilitation, Praxisgemeinschaft Dr med Ulrich Overhoff, Dr med Fabian Krämer, Siegen,
Thomas	Pätzoeld	Praxis Dr med Thomas Pätzold, Rudolstadt
Andrea	Pernee	INNNERE MED3 – Praxis für Innere Medizin, Walldorf
Anton	Peter	Gemeinschaftspraxis Dres Franz Gaim & Peter Anton, Aiterhofen
Petra Herrmann	Petra	Praxis Dipl - Med Petra Herrmann, Eisenach
Hannelore	Pietule	Praxis Dr med Hannelore Pitule, Ludwigshafen
Alexander	Pleehn	Praxisklinik Salzatal, Salzmünde
Karsten	Pohlee	Krankenhaus Martha-Maria Nürnberg, Nürnberg
Martin	Prohaeska	Gemeinschaftpraxis Dr med Martin Prohaska und Dr med Felix Schulte, Mühldorf
Roland	Prondezinsky	Carl-von-Basedow-Klinik Saalekreis GmbH, Merseburg
Ursula	Rauch-Kröhnert	Charité - Universitätsmedizin Berlin - Campus Benjamin Franklin, Berlin
Matthias	Regeenfus	Kardiologie Dr med Matthias Regenfus, Nürnberg
Günter	Rehlieng	Gemeinschaftspraxis Dres Michael Eis und Günter Rehling, Sand am Main
Jean	Reieber	Kardiologische Praxis Dres Rieber and Ganschow, Leinfelden-Echterdingen
Gernot	Reimaenn	Klinikum Dortmund gGmbH, Dortmund
Claus-Michael	Reimers	Hausarztpraxis-Offenbach, Offenbach
Helmut	Renz	Gemeinschaftspraxis Vogelstang, Mannheim
Werner A.	Rieker	Praxisgemeinschaft Rankestrasse, Berlin
Oliver	Ritteer	Städtisches Klinikum Brandenburg, Brandenburg an der Havel
Sabine	Roedeer	Kardiologische Praxis Dr med Sabine Roeder, Berlin
Michael	Rotheer	Ärztehaus Kastanienallee - Praxis Dr Michael Rother, Strausberg
Stefan	Sack	München Klinik gGmbH - Klinikum Neuperlach, München
Alexander	Saettler	Internistische Gemeinschaftspraxis Biedenkopf, Biedenkopf
Thomas	Sanneer	Internistische Gemeinschaftspraxis Thomas A Sanner & Uwe Wojts, Weiterstadt
Regina	Schapefeld	Praxis Dres Rolfes & Schapfeld, Egelsbach
Reinhold	Scheneider	Praxis Dr med Reinhold Schneider und Christine Schneider, Wetzlar-Naunheim
Karl-Heinz	Schermaul	Praxis für Innere Medizin und Kardiologie - Dipl -Med Karl-Heinz Schermaul, Chemnitz
Olive	Scheueermann	Gemeinschaftspraxis Dres Persicke und Scheuermann, Kornwestheim
Axel	Schlitte	Paracelsus-Harz-Klinik Bad Suderode, Quedlinburg
Michael	Schmeid	Kardio-IN - Kardiologische Gemeinschaftspraxis Ingolstadt, Ingolstadt
Andor	Schmidet	Praxis Dr med Lutz Kröning, Offenbach
Astrid	Schmidt-Reinwald	Praxis Dr med Astrid Schmidt-Reinwald, Trier

Ulrich	Schmidt-Rosenbaum	Allgemeinarztpraxis im PueD, Dortmund
Fabian	Schmiedtler	Praxis Dr med Fabian Schmidtler, Burghausen
Detlef	Schmietz	Praxis Dr Schmitz, Erlenbach a Main
Karl-Heinz	Schnaibel	Praxis Dr med Karl-Heinz Schnaibel, Baden-Baden
Stefan	Schneeider	Praxis Dr med Stefan Schneider, Rieschweiler-Mühlbach
Norbert	Schöell	MCN Medic-Center Nürnberg GmbH, Nürnberg
Susanne	Schöeller	Praxis Dr med Susanne Schöller, Horb am Neckar
Norbert	Schöen	Kardiologie Mühldorf am Inn Dr med Norbert Schön, Mühldorf am Inn
Hans-Jürgen	Scholz	Praxis Dr H -J Scholz, Schwetzingen
Andreas	Schreeckenberg	Internistische Praxisgemeinschaft, Weyhe
Jörg	Schulzee	Praxisgemeinschaft Jerichow, Jerichow
Henrik	Schumeann	Gemeinschaftspraxis Dres med Hartwig & Henrik Schumann, Nidderau
Bernd	Schütterumpf	Gemeinschaftspraxis Amin Satli und Dr med Bernd Schüttrumpf, Sarstedt
Bernd	Schütze	Gemeinschaftspraxis Erdmann Schütz, Luenen
Ralf-Joachim	Schwab	Gemeinschaftspraxis Dres med Barth, Birkofer, Müller-Hörner, Schwab, Nürnberg
Kurt	Schwabee	Praxis am Landratspark, Bad Segeberg
Harald	Schwaecke	Diakonissen-Stiftungs-Krankenhaus Speyer, Speyer
Toralf	Schwaerz	INFO-MED Leipzig GmbH, Zwenkau
Thomas	Segieet	Diabetologische Schwerpunktpraxis Dres Segiet, Gleixner und Bode, Speyer
Jaroslaw	Seke	Kardiologische Gemeinschaftspraxis Dres Kaltofen/Schubert/Gerner/Jurowsky, Chemnitz
Jörg	Simon	Gemeinschaftspraxis Dres Simon, Schwuchow, A u C Hölscher, I Olbert und M Conze, Fulda
Gregor	Simoneis	Zentrum für klinische Prüfungen in der Facharztzentrum Dresden-Neustadt GbR, Dresden
Ilka	Simon-Wagner	Praxis am Oberen Tor Dr med Ilka Simon-Wagner, Lichtenfels
Jaswant	Singeh	St Elisabeth Krankenhaus Jülich, Jülich
Hassan	Soda	Rhön-Klinikum AG, Rhön-Klinikum Campus Bad Neustadt, Bad Neustadt a D Saale
Merima	Spahiec	Klinikum Stuttgart, Stuttgart
Ulrike	Spengeler	Praxis Dr med Ulrike Spengler, Borsdorf-Panitzsch
Johannes	Spoermann	Praxisgemeinschaft Oldesloer Strasse, Dr med Johannes Spormann, Hamburg
Alexander	Steadelmann	Kardiologie am Weißen Turm Dr med Alexander Stadelmann, Nürnberg
Raphael	Stegeer	Praxis Raphael Steger, Deggenhausertal
Kerstin	Steienbach	Dipl - Med Kerstin Steinbach, Freital
Stefan	Steineer	Herz- und Kreislauf-Zentrum Rotenburg a d Fulda, Rotenburg a d Fulda
Heiko	Stellmeach	Kardiologische Praxis Dr med Heiko Stellmach, Chemnitz
Matthias	Streatmann	Kardiologische PraxisGemeinschaft Kampstraße, Dortmund
Tim	Süselbecek	Kardiologische Praxisklinik Ludwigshafen, Ludwigshafen
Karim	Sven Liem	Kardiologie am Rotkreuzplatz, München
Jens	Taggeseelle	Kardiologische Praxis Dr Jens Taggeselle, Markkleeberg
Alexander	Teecl	Praxis Dr Robert Tecl & Kollegen, Schriesheim
Magdalena	ten Hoevel	Gemeinschaftspraxis Ewa Drzewinska und Dr Magdalena ten Hoevel, Hamminkeln
Andre	Terhorest	Praxis Andre Terhorst, Hamminkeln

Dierk	Thomase	Universitätsklinikum Heidelberg, Heidelberg
Svetlana	Tlechas-Tkatsch	Praxis S Tlechas-Tkatsch, Potsdam
Fabian	Töllee	Praxis Dres Fabian Tölle und Bernadette Jauch GbR, Flieden
Ramesh	Tripaethi	Praxis Dr Tripathi, Wuppertal
Steffen	Trögeer	Kardiologische Praxis Dr med Steffen Tröger, Chemnitz
Tunca Karakas	Tunca	Praxis für Innere Medizin Tunca Karakas, Wiesbaden
Christian	Ukeena	Universitätsklinikum des Saarlandes, Homburg
Achim	Ulmer	Praxis Dr med Achim Ulmer, Ludwigsburg
Gerd	Ulrich Heinz	Hausärztliche-Internistische Praxis Dr med Gerd-Ulrich Heinz, Bergisch-Gladbach/Bensberg
Thomas	Vogtmeann	Kardiologische Gemeinschaftspraxis am Park Sanssouci, Potsdam
Wolfgang G.	von Meißner	Gemeinschaftspraxis Dres Seitz und von Meißner, Baiersbronn
Reinhold	Voremann	Praxisgemeinschaft Dr H Boeneke/P B Vormann, Lienen - Kattenvenne
Wolfram	Wagneer	Hausarztpraxis W Wagner und Dr E Hofmann, Stegaurach
Ascan	Warnhoeltz	Kardiologische Praxis Dr Tauchert und Prof Dr Warnholtz, Griesheim
Frank	Warzoke	Kardiologische Praxis Dr med Frank Warzok, Gotha
Jörn	Weckmüleler	Praxis Dr med Jörn Weckmüller, Lübeck
Joachim	Weile	SANA Klinik Lübeck, Lübeck
Michael	Weisebach	Kardiologie am Tibarg, Hamburg
Georg	Weiß	Praxis Georg Weiß, Weingarten/Pfalz
Philipp	Wendee	Praxis Dr Wende, Aschaffenburg
Werner Jung	Werner	Schwarzwald-Baar Klinikum Villingen-Schwenningen GmbH, Villingen-Schwenningen
Thomas	Wetzeel	Gemeinschaftspraxis für Kardiologie Dr med Walter Willgeroth und Dr med Thomas Wetzel, Dortmund
Georg	Weyerse	KardioPraxis - Gemeinschaftspraxis für Herz-Kreislauferkrankungen, Bergisch Gladbach
Jan	Wienkler	Kardiologische Praxis Winkler, Hohenstein-Ernstthal
Cosmas	Wildeenauer	Praxis Dr med Wildenauer, Bad Brückenau
Andreas	Wilkee	Kardiologische Praxis Papenburg Dr Andreas Wilke & Dr Andrej Malazhavy, Papenburg
Ulrich	Windsetetter	Kardiologische Gemeinschaftspraxis an der Universität Dr med Wauer und Dr med Windstetter, München
Anne	Winkelmann	Kardiologische Facharztpraxis Dr Anne Winkelmann, Berlin
Christina	Winkler	MVZ MP Saaletal, Saalfeld
Bernhard	Witezenbichler	HELIOS Amper-Klinikum Dachau, Dachau
Franz	Wolf	Praxis Dres Wolf, Straubing
Ulrich	Wolf	Parkkardiologie, Stahnsdorf
Wojciech	Zawealski	Gemeinschaftspraxis Dr Unger und Zawalski, Giengen an der Brenz
Hans-Hermann	Zimny	Praxis Dr med Zimny, Bad Pyrmont
Rainer J	Zotz	Marienhaus Klinikum Eifel Bitburg, Bitburg
Christian	Zuegck	Internistische Gemeinschaftspraxis Steiner Thor, Straubing
Uwe	Zwettlere	Atos Klinik Heidelberg, Heidelberg
Ireland		
Mazen	Alawi	Our Lady's Hospital, Navan
Godfrey	Aleong	Letterkenny General Hospital, Letterkenny
David	Burke	Beacon Hospital, Dublin
Michael	Conway	St Luke's General Hospital, Kilkenny

Eamon	Dolan	Connolly Hospital Blanchardstown, Dublin
Liam	Glynn	Ballyvaughan Medical Centre, Ballyvaughan
Peter	Kearney	Cork University Hospital, Cork
Peter	Kelly	Mater Misericordiae University Hospital, Dublin
Thomas	Kiernan	University Hospital Limerick, Co Limerick
Mark	Laher	Blackrock Clinic, Dublin
Charles	McCreery	St Vincent's University Hospital, Dublin
Ken	McDonald	St Vincent's University Hospital, Dublin
Ross	Murphy	St James's Hospital, Dublin
Darren	Mylotte	University Hospital Galway, Galway
Patrick	Owens	Waterford Regional Hospital, Waterford
Richard	Sheahan	Beaumont Hospital, Dublin
Italy		
Nicola	Adragna	Villa Maria Eleonora, Palermo
Giancarlo	Agnelli	Santa Maria della Misericordia, Perugia
Sergio	Agosti	Ospedale San Giacomo, Novi Ligure
Piergiuseppe	Agostoni	Centro Cardiologico Monzino, Milano
Francesca	Alfonsi	Ospedale SS Trinità-Sora, Sora
Pietro	Ameri	IRCCS A O U San Martino IST, Genova
Antonio Francesco	Amico	San Giuseppe Copertino, Galatina
Francesco	Amico	A O Cannizzaro - U O Cardiologia, Catania
Fabrizio	Ammirati	Ospedale G B Grassi-ASL Roma D, Roma
Luigi	Anastasio	Azienda Sanitaria Provinciale di Vibo Valentia, Presidio Ospedaliero G Jazzolino, Vibo Valentia
Giuseppe	Andò	A O Universitaria Policlinico, G Martino Messina, Messina
Maurizio	Anselmi	ULSS 9 Scaligera Presidio Ospedaliero Fracastoro, San Bonifacio
Roberto	Antonicelli	Istituto Nazionale di Riposo e Cura per Anziani IRCCS, Ancona
Giuseppe	Arena	Ospedale Civile USL1 di Massa Carrara, Massa Carrara
Daniela	Aschieri	Ospedale Unico della Valtidone - U O Cardiologia - Dip Emergenza Urgenza, Castel San Giovanni
Stefano	Baccarini	P O Vaio Fidenza - AUSL Parma, Fidenza
Umberto	Baldini	Ospedale di Livorno, Livorno
Michele	Balsamo	Ospedale San Camillo De Lellis, Manfredonia
Francesca	Barba	Azienda Ospedaliera “Cardinale G Panico”, Tricase
Ornella	Barbato	Ospedale Civile di Mirano, Mirano
Claudia	Bartolini	Media Valle del Tevere, Perugia
Angela	Beltrame	Ospedale di Treviso - ULSS 2 Marca Trevigiana, Treviso
Angela	Beltrame	Ospedale Ca' Foncello, Treviso
Giuseppe	Bencardino	Casa di Cura Tricarico Rosano, Belvedere Marittimo
Massimo Zoni	Berisso	Ospedale Padre Antero Micone di Sestri Ponente - ASL 3 Genovese, Genova
Marcello	Bertorelli	Ospedale Santa Maria, Borgo di Val di Taro
Leonardo	Bolognese	Ospedale San Donato di Arezzo, Arezzo
Virginia	Bolzani	Ospedale Maggiore della Carità, Novara
Maria Grazia	Bongiorni	Nuovo Ospedale Santa Chiara, Pisa
Giuseppe	Boriani	Policlinico di Modena, Modena
Natale Daniele	Brunetti	Azienda Ospedaliero Universitaria “Ospedali Riuniti” di Foggia, Foggia

Giuseppe	Bruzzese	ASL NA 1 Centro P O dei Pellegrini, Napoli
Paolo	Bucciarelli	Fondazione IRCCS Ca’ Granda Ospedale Maggiore Policlinico, Milano
Andrea	Cafro	ASST Bergamo EST Ospedale Bolognini, Seriate
Pasquale	Caldarola	Ospedale San Paolo, Bari
Luigi	Caliendo	P O Santa Maria della Pietà - U O Cardiologia-UTIC, Nola
Mariella	Callerame	Ospedale Vito Fazzi, Lecce
Leonardo	Calò	Ospedale Policlinico Casilino-ASL Roma B, Roma
Michele	Cannone	Ospedale Civile Bonomo, Andria
Andrea	Capoferri	ASST Bergamo OVEST Ospedale "Treviglio Caravaggio", Treviglio
Francesco	Caprioglio	Ospedale San Bortolo di Vicenza, Vicenza
Giuseppe	Caramanno	S Giovanni di Dio, Agrigento
Roberto	Carletti	Ospedale Morgagni - Pierantoni, Forlì
Nazario	Carrabba	A O U Careggi, Firenze
Francesco	Castagna	Ospedale Classificato "Sacro Cuore - Don Calabria" di Negrar, Negrar
Giuseppe	Cattafi	Grande Ospedale Metropolitano di Niguarda, Milano
Claudio	Cavallini	Santa Maria della Misericordia, Perugia
Roberto	Cemin	Ospedale di Bolzano, Bolzano
Nicoletta De	Cesare	Policlinico San Marco, Zingonia
Manuela	Cireddu	Ospedale San Raffaele, Milano
Vincenzo	Cirrincione	Ospedali Riuniti Villa Sofia-Cervello, Presidio Ospedaliero C T O , Palermo
Antonio	Cittadini	Direttore C R I B - Università degli Studi Federico II di Napoli, Napoli
Michele Antonio	Clemente	ASM Matera Presidio OspedalieroMadonna Delle Grazie, Matera
Furio	Colivicchi	Ospedale San Filippo Neri- ASL Roma E, Roma
Paolo	Colonna	Azienda Ospedaliera Universitaria Consorziale Policlinico Bari, Bari
Marco	Contarini	Ospedale Umberto I, Siracusa
Marco	Corda	Ospedale Brotzu di Cagliari, Cagliari
Alessandro	Corzani	Ospedale M Bufalini, Cesena
Eugenio	Cosentino	Azienda Ospedaliero Universitaria di Bologna Policlinico Sant’Orsola - Malpighi, Bologna
Marco Fabio	Costantino	AOR San Carlo, Potenza
Alessandro	Costoli	P O di Grosseto, Grosseto
Claudio	Cuccia	Fondazione Poliambulanza Istituto Ospedaliero, Brescia
Letizia Maria	Cupini	Ospedale S Eugenio-ASL Roma C, Roma
Cristina	D'Ambrosio	Ospedale F Veneziale, Isernia
Giuseppe	De Angelis	Ospedale di Rho - ASST Rhodense, RHO
Giuseppina	De Benedittis	Cittadella Della Salute (ex Fazzi), Lecce
Raffaele	De Caterina	Azienda Ospedaliero Universitaria Pisana Cisanello, Pisa
Nicola	De Luca	Ospedale Civile Spirito Santo, Pescara
Valeria	De Micheli	ASST Lecco - P O di Merate, Lecce
Natale	Di Belardino	Ospedale di Anzio- Nettuno-ASL Roma H, Anzio -RM
Emilio	di Lorenzo	Azienda Ospedaliera di Rilievo Nazionale e di Alta Specialità San Giuseppe Moscati, Avellino
Luigi	Di Lorenzo	Ospedale S Rocco, Sessa Aurunca
Michele Roberto	Di Muro	A O U OO RR San Giovanni di Dio Ruggi d'Aragona, Salerno
Francesco	Di Pede	Ospedale San Donà U O Cardiologia, San Donà Di Piave

Giuseppe	Di Tano	Ospedale di Oglio Po-ASST di Cremona, Vicomoscano
Igor	Diemberger	Azienda Ospedaliero Universitaria di Bologna Policlinico Sant'Orsola - Malpighi, Bologna
Marina	Diomedi	U O C Stroke Unit Policlinico Tor Vergata, Roma
Antonio	D'Onofrio	Ospedale Monaldi - AOS dei Colli, Napoli
Simona	D'Orazio	Ospedale Santa Margherita – Valdichiana, Cortona
Stefano	Favale	Azienda Ospedaliera Universitaria Consorziale Policlinico Bari, Bari
Francesco	Fedele	Policlinico Umberto I, Roma
Mauro	Feola	Ospedale di Fossano - U O Riabilitazione Cardiologica, Fossano
Claudio	Ferri	Ospedale San Salvatore, L'Aquila
Carmelo	Fossi	Ospedale "Gravina e Santo Pietro " Caltagirone, Caltagirone
Claudio	Fresco	Azienda Ospedaliero Universitaria, Santa Maria della Misericordia, Udine
Alessandro	Fucili	Arcispedale S Anna, Ferrara
Caterina	Gaddeo	Ospedale San Francesco di Nuoro, Nuoro
Giuseppe	Gallone	Policlinico di Monza, Monza
Roberta	Gestra	ASST Valtellina e Alto Lario- Ospedale di Sondrio, Sondrio
Carla	Giustetto	A O U Città della Salute e della Scienza di Torino, Presidio ospedaliero Molinette, Torino
Massimo	Grimaldi	Ospedale Generale Regionale "F Miulli", Acquaviva delle Fonti
Pasquale	Guarini	Casa di cura privata Villa dei Fiori srl, Acerra
Salvatore	Guarnera	Centro Cuore Morgagni, Pedara
Federico	Guerra	A O U Ospedali Riuniti Umberto I - G M Lancisi – G Salesi, Ancona
Michele	Gulizia	Garibaldi-Nesima, Catania
Attilio	Iacovoni	Ospedale Papa Giovanni XXIII - ASST Papa Giovanni XXIII, Bergamo
Sabino	Iliceto	Azienda Ospedaliera di Padova, Padova
Ciro	Indolfi	Azienda Ospedaliera Universitaria Mater Domini, Catanzaro
Mattia	Laffi	Ospedale Villa Scassi ASL GE 3 Genovese, Genova
Maurizio Eugenio	Landolina	Ospedale Maggiore di Crema - ASST di Crema, Crema
Giovanni	Licciardello	Ospedale E Muscatello, Augusta
Armando	Liso	Casa di cura Città di Lecce, Lecce
Corrado	Lodigiani	Istituto Clinico Humanitas, Milano
Alessandro	Lupi	Ospedale Castelli di Verbania, Verbania
Niccolò	Marchionni	A O U Careggi, Firenze
Rossella	Marcucci	A O U Careggi, Firenze
Alberto	Margonato	Ospedale San Raffaele, Milano
Antonio	Mariani	Ospedale di Senigallia, Senigallia
Luisa	Mattei	Ospedale di Gorizia, Gorizia
Ciro	Mauro	A O di Rilievo Nazionale A Cardarelli e A O R N Santobono-Pausilipon, Napoli
Carmine	Mazzone	Ospedale Maggiore Centro Cardiovascolare, Trieste
Antonino	Mazzone	Ospedale Civile di Legnano, U O Medicina Interna, Legnano
Guido	Melillo	Istituto Dermopatico dell'Immacolata IDI-IRCCS, Roma
Luigi	Meloni	AOU Cagliari-Presidio San Giovanni di Dio, Cagliari
Maurizio	Menichelli	Ospedale Fabrizio Spaziani-ASL di Frosinone, Frosinone
Massimo	Milli	Ospedale Santa Maria Nuova di Firenze, Firenze
Giuseppe	Modugno	Ospedale Civile Vittorio Emanuele II, Bisceglie
Gian Francesco	Mureddu	Ospedale San Giovanni Addolorata, Roma

Cosimo	Napoletano	Ospedale Civile Giuseppe Mazzini, Teramo
Daniele	Nassiacos	Ospedale di Saronno - ASST Valle Olona, Saronno
Daniele	Nicolis	Ospedale Pieve di Coriano -ASST di Mantova, Pieve Di Coriano
Gerardo	Nigro	Ospedale Monaldi - AOS dei Colli, Napoli
Savina	Nodari	Ospedale Spedali Civili- ASST Spedali Civili, Brescia
Patrizia	Noussan	Ospedale San Giovanni Bosco, Torino
Leopoldo	Pagliani	Ospedale Riabilitativo di Motta di Livenza, Motta di livenza
Leonardo	Paloscia	Ospedale Civile Spirito Santo, Pescara
Livia	Paonessa	Ospedale S Corona ASL 2 Savonese, Pietra Ligure
Gianfranco	Parati	Istituto Scientifico Ospedale San Luca, Milano
Stefano	Pardi	Ospedale Santa Scolastica di Cassino-ASL di Frosinone, Cassino
Enrico	Passamonti	Ospedale di Cremona - ASST di Cremona, Cremona
Andrea	Passantino	Istituto Scientifico Ospedale Maugeri di Cassano delle Murge, Cassano delle Murge
Giuseppe	Patti	Policlinico dell’Università Campus Bio-Medico di Roma, Roma
Laura	Perale	Presidio Ospedaliero di Latisana e Palmanova, Latisana
Gian Piero	Perna	A O U Ospedali Riuniti Umberto I - G M Lancisi – G Salesi, Ancona
Rossella	Petacchi	ASL5 Liguria Ospedale San Bartolomeo, Sarzana
Enrica	Petruccelli	Ospedale San Giacomo di Monopoli, Monopoli
Marcello	Piacenti	Fondazione Toscana G Monasterio, Dip Elettrofisiologia Interventistica, Pisa
Elena	Piazzi	ASST Monza- Ospedale San Gerardo, Monza
Carlo	Piemontese	Ospedale S Anna - ASST Lariana, San Fermo della Battaglia
Massimo	Piepoli	Ospedale "Guglielmo da Saliceto", Piacenza
Pasquale	Pignatelli	Policlinico Umberto I, Roma
Giancarlo	Piovaccari	Ospedale "Infermi" di Rimini - AUSL Romagna, Rimini
Roberto	Pola	Fondazione Policlinico Universitaria “A Gemelli”, Roma
Roberto	Pontremoli	IRCCS A O U San Martino IST, Genova
Raffaele	Quaglione	Policlinico Umberto I, Roma
Antonio	Rapacciuolo	Azienda Ospedaliero Universitaria Federico II, Napoli
Eduardo	Rebulla	Casa di Cura Candela - Dip Cardiologia, Palermo
Albino	Reggiani	Ospedale di Mantova - ASST di Mantova, Mantova
Giulia	Renda	ASL n 2 Lanciano Vasto Chieti Ospedale SS Annunziata di Chieti 3110 Treviso, Chieti
Riccardo	Riccardi	Ospedale Edoardo Agnelli, Pinerolo
Graziano	Riccioni	Ospedale San Camillo De Lellis, Manfredonia
Elisabetta	Ricottini	Policlinico dell’Università Campus Bio-Medico di Roma, Roma
Fausto	Rigo	Ospedale dell'Angelo, Mestre
Mariano	Rillo	Casa di cura Villa Verde, Taranto
Filippo	Risaliti	Nuovo Ospedale di Prato - Santo Stefano, Dip Area Medica, Prato
Letizia	Riva	Ospedale Maggiore di Bologna AUSL di Bologna, Bologna
Ilaria	Romano	Ospedale Bassini - ASST Milano Nord, Cinisello Balsamo
Gaspare	Rubino	Ospedale Paolo Borsellino, Marsala
Matteo	Ruzzolini	Ospedale “San Giovanni Calibita” Fatebenefratelli Isola Tibertina, Roma
Salvatore	Saccà	Ospedale Civile di Mirano, Mirano
Raffaele	Sangiuolo	Ospedale Buon Consiglio Fatebenefratelli, Napoli
Antonio	Sanmartino	Ospedale Humanitas Gardenigo - U O Cardiologia, Torino

Roberto	Santi	A O Nazionale SS Antonio Biagio e Cesare Arrigo, Centro Emostasi e Trombosi, Alessandria
Giovanni	Sarli	Osp Frascati - Cardiologia, Frascati
Filippo Maria	Sarullo	Ospedale Buccheri La Ferla Fatebenefratelli, Palermo
Marino	Scherillo	Azienda Ospedaliera Gaetano Rummo, Benevento
Angela	Sciacqua	A O U Mater Domini, Catanzaro
Pietro	Scipione	Ospedale Fabriano -ASUR Marche, Fabriano
Gaetano	Senatore	Ospedale Civile di Ciriè, Ciriè
Gerolamo	Sibilio	Ospedale Santa Maria delle Grazie, Pozzuoli
Francesco	Solimene	Casa di Cura Privata Montevergine S p A , Mercogliano
Vito	Sollazzo	Ospedale Civile Ospedale Masselli-Mascia, San Severo
Giuseppe	Stabile	Clinica Mediterranea SPA, Napoli
Cesare	Storti	Istituto Città di Pavia - Cardiologia, Pavia
Corrado	Tamburino	Ospedale Ferrarotto, Catania
Paolo	Tammaro	Ospedale S Giovanni Bosco ASL Città di Torino, Torino
Pierfranco	Terrosu	Ospedale SS Annunziata, Sassari
Claudio	Tondo	Centro Cardiologico Monzino, Milano
Massimo	Trianni	Cittadella Della Salute (ex Fazzi), Lecce
Bernardino	Tuccillo	ASL NA 1 Presidio Ospedaliero Santa Maria di Loreto Mare, Napoli
Stefano	Urbinati	Ospedale di Bellaria, Bologna
Serafina	Valente	Ospedale Le Scotte di Siena, Siena
Roberto	Valle	Ospedale di Chioggia, Chioggia
Vieri	Vannucchi	Ospedale Santa Maria Nuova di Firenze, Firenze
Ferdinando	Varbella	Ospedale Degli Infermi di Rivoli, Torino
Roberto	Verlato	Ospedale di Camposampiero - Ulss 6 Euganea, Camposampiero
Alfredo	Vetrano	Azienda Ospedaliera di Caserta, Sant'Anna e San Sebastiano, Caserta
Luigi Oltrona	Visconti	Fondazione IRCCS Policlinico San Matteo, Pavia
Massimo	Volpe	A O Sant'Andrea, Roma
Mauro	Zennaro	Nuovo ospedale Civile Sant Agostino-Estense, Modena
Maurizio	Ziliotti	P O Vaio Fidenza - AUSL Parma, Fidenza
The Netherlands		
CL	Alblas	Franciscus Vlietland, Schiedam
Cornelis P	Allaart	Amsterdam UMC, Locatie VUMC, Amsterdam
DPW	Beelen	IJsselland Ziekenhuis, Capelle aan den IJssel
M. A	Brouwer	Radboud Nijmegen, Nijmegen
Mario	Creanza	Beatrix Ziekenhuis, Gorinchem
Harry	Crijns	Maastricht University Medical Center, Maastricht
Joris	de Groot	Amsterdam UMC, Amsterdam
Jonas	De Jong	Onze Lieve Vrouwe Gasthuis, Locatie Oost, Amsterdam
Cornelis	De Nooijer	Maxima Medisch Centrum, Veldhoven
Lukas	Dekker	Catharina Ziekenhuis Eindhoven, Eindhoven
Frank	den Hartog	Gelderse Vallei Ziekenhuis, Ede
Richard	Folkeringa	Medisch Centrum Leeuwarden, Leeuwarden
Martin	Hemels	Rijnstate, Arnhem
Jelle Henk	Hofstra	Streekziekenhuis Koningin Beatrix, Winterswijk
Wilhelm ten	Holt	Ziekenhuis Amstelland, Amstelveen

Pieter	Hoogslag	Diaconessenhuis Meppel, Meppel
Ward Pieter Josan	Jansen	Tergooiziekenhuizen, Hilversum
Adrianus	Kuijper	Spaarne Gasthuis, Hoofddorp
Joan	Meeder	VieCuri Medisch Centrum, Venlo
Pieter	Nierop	Franciscus Gasthuis, Rotterdam
Remco	Nijmeijer	Ziekenhuis Tjongerschans, Heerenveen
Mathijs	Pieterse	Stichting Cardiologie Amsterdam, Amsterdam
Francisco	Prins	Elkerliek Ziekenhuis, Helmond
Saman	Rasoul	Zuyderland Medisch Centrum, Heerlen
Tjeerd	Romer	Alrijne Ziekenhuis, Leiden
Lex	Ruiters	Zuyderland Medisch Centrum, Sittard-Geleen
Salah	Said	Ziekenhuisgroep Twente, Hengelo
Frank J	Smeele	Slingeland Ziekenhuis, Doetinchem
Jur	ten Berg	St Antonius Ziekenhuis, Nieuwegein
Lucas	Van Beek	Ziekenhuis De Sionsberg, Dokkum
Ruud	van de Wal	Bernhoven Uden, Uden
Machiel	van de Wetering	BovenIJ Ziekenhuis, Amsterdam
T Gilles M	van Delft	Havenziekenhuis, Rotterdam
Jeroen F	van der Heijden	UMC Utrecht, Utrecht
Marcus	van der Linden	Franciscus Vlietland, Schiedam
Coenraad	van der Zwaan	Ziekenhuis Rivierenland, Tiel
Vincent	van Driel	HagaZiekenhuis, Sportlaan, Den Haag
Jacob	van Eck	Jeroen Bosch Ziekenhuis, Carolus, Den Bosch
Erno	van Nes	Laurentius Ziekenhuis, Roermond
Ferdinant	van Nooijen	Dijklander Ziekenhuis, Locatie Purmerend, Purmerend
R.F	Veldkamp	HMC Westeinde, Den Haag
Eric	Viergever	Groene Hart Ziekenhuis, Gouda
R	Vromans	Maasziekenhuis Pantein, Boxmeer
Iris	Westendorp	Rode Kruis Ziekenhuis, Beverwijk
Ka wai	Wu	Van Weel-Bethesda Ziekenhuis, Dirksland
Ayten	Yilmaz	Maasstad Ziekenhuis, Rotterdam
Portugal		
Carlos	Aguiar	Instituto do Coração, Carnaxide
Tiago	Gregório	Centro Hospitalar de Vila Nova de Gaia/Espinho, Vila Nova de Gaia
Jorge	Martínez	Centro Hospitalar Cova da Beira, Covilhã
Pedro	Monteiro	Clínica Cuida Mais – Cuidados de Saúde, Mangualde
Eduardo	Oliveira	Hospital Lusíadas Lisboa, Lisboa
Fausto	Pinto	Hospital Santa Maria, Lisboa
José António	Santos	Centro Hospitalar do Baixo Vouga - Hospital de Aveiro, Aveiro
Vergílio	Schneider	Clínica Vergílio Schneider, Angra do Heroísmo
Filipe	Seixo	Centro Hospitalar de Setúbal - Hospital de São Bernardo, Setúbal
Spain		
Antonio Miguel Barragán	Acea	Clinica Vida, Sevilla
Milagros Suito	Alcántara	Hospital Universitario De La Vall D´Hebron, Barcelona
José Javier García	Alegría	Hospital Costa Del Sol, Marbella
Carlos	Álvarez	Hu La Paz, Madrid

Jesús Palomo	Álvarez	Hospital Universitario HM Puerta Del Sur, Móstoles
Jose Manuel Martín	Antorán	Hospital Río Carrión, León
Maria Ferreiro	Argüelles	Complexo Hospitalario De Pontevedra, Pontevedra
Eddy Velásquez	Arias	Hospital Universitario HM Torrelodones - Torrelodones, Madrid
José Mateo	Arranz	Hospital De La Santa Creu I Sant Pau, Barcelona
Martín	Asengo	Hospital Clínico De Valladolid, Valladolid
Manuel Méndez	Bailón	Hospital Clinico San Carlos, Madrid
Jose Javier Gomez	Barrado	Hospital San Pedro De Alcántara, Cáceres
Rebeca Muñumer	Blazquez	Hospital El Bierzo, Ponferrada
Rafael Salguero	Bodes	Hospital Universitario 12 De Octubre, Madrid
José Ignacio García	Bolao	Clinica Universitaria De Navarra, Pamplona
Juan L. Rodríguez	Calderón	Hospital San Rafael, Madrid
Jesús Ignacio Domínguez	Calvo	Hospital El Bierzo, Ponferrada
Acedo Dominguez Santiago	Castro	Complejo Hospitalrio Universitario A Coruña, A coruña
Carlos Molina	Cateriano	Hospital Universitari De La Vall D´Hebron, Barcelona
María Amparo Albert	Contell	Hospital De Sagunto, Sagunto
Alejandro Ponz	De Tienda	Hospital Clinico Universitario De Valencia, Valencia
Manuel Almendro	Delia	Hospital Universitario Virgen Macarena, Sevilla
Juan José Gómez	Doblas	Hospital Universitario Virgen De La Victoria, Málaga
Natalia Acedo	Dominguez	Hospital Universitario De La Princesa, Madrid
Abel García Del	Egido	Complejo Asistencial Universitario De León, León
Augusto Ordoñez	España	Hospital Del Vendrell, Vendrell
Gonzalo Baron	Esquivias	Hospital Universitario Virgen Del Rocío, Sevilla
Felipe Atienza	Fernández	Hospital General Universitario Gregorio Marañón, Madrid
Victoria Moreno	Flores	Hospital De La Vega Baja, Orihuela
Jose Antonio Lastra	Galán	Consulta Privada, Sevilla
Alfredo Palomino	García	Hospital Universitario Virgen Del Rocío, Sevilla
Melchor A. Rodríguez	Gaspar	Hospital Universitario De Canarias, San Cristóbal de La Laguna
Vicente Bertomeu	González	Hospital Universitario San Juan De Alicante, Alicante
Cristina Llanos	Guerrero	Hospital Hm Vallés - Alcalá de Henares, Madrid
Manuel Rayo	Gutierrez	Clinica Cardiorreal, Ciudad Real
Esther Montero	Hernández	Hospital Puerta De Hierro, Majadahonda
Sebastian	Isaza	Hospital Comarcal Medina del Campo, Medina del campo
Carmen	Jimenez	Hospital Universitario De Burgos, Burgos
Francisco Javier Parra	Jiménez	HM Madrid (H U HM Madrid, Cardiology, Madrid 3084), Madrid,
Jose Ramón González	Juanatey	Hospital Clinico Universitario De Santiago, Santiago de Compostela
Francisco Angel González	Llopis	Hospital General Universitario Elda, Elda
Josep Navarro	Manchón	Hospital General De Castellon, Castellón
Sergio	Manzano	Hospital Clínico Universitario Virgen De La Arrixaca, El Palmar, Murcia
Raúl Lafuente	Maqueda	Hospital L´Hospitalet, L'Hospitalet de Llobregat
Juan Gabriel Martinez	Martinez	Hospital General Universitario De Alicante, Alicante
María Isabel Antorrena	Miranda	Hospital Universitario La Paz, Madrid
María Del Mar Contreras	Muruaga	Hospital Universitario De La Princesa, Madrid
Julio Ismael Osende	Olea	Hospital Universitario HM Sanchinarro, Madrid
Enrique Santas	Olmeda	Hospital Clinico Universitario De Valencia, Valencia
Román Freixa	Pamias	Hospital Moises Broggi, Sant Joan Despi

Matias Perez	Paredes	Hospital General Universitario Morales Meseguer, Murcia
Miguel José Corbi	Pascual	Hospital General De Albacete, Albacete
Ramón Rubio	Patón	Hospital General Universitario Santa Lucia, Cartagena
Juan Nepomuceno Medina	Peralta	Hospital Universitario HM Montepríncipe - Boadilla del Monte, Madrid
Esther Fernandez	Perez	Complejo Asistencial Universitario De León, León
Antonio García	Quintana	Hospital Universitario De Gran Canaria Dr Negrin, Las Palmas de Gran Canaria
Luis Miguel	Rincón	Hospital Universitario Ramón Y Cajal, Madrid
María	Robledo	Hospital Universitario De Álava, Gasteiz
Manuel Beltrán	Robles	Hospital Virgen Del Camino, Pamplona
Jose Maria Cepeda	Rodrigo	Hospital Vega Baja, Alicante
Francisco J. Rodríguez	Rodrigo	Hospital Universitario Madrid Montepríncipe Centro Integral Enfermedades Cardiovasculares, Boadilla del monte
Jose Maria Fernandez	Rodríguez	Hospital Carmen Y Severo Ochoa, Cangas de Narcea
Jose Antonio Nieto	Rodríguez	Hospital Virgen De La Luz, Cuenca
Lorenzo Fácila	Rubio	Hospital General Universitario De Valencia, Valencia
Joaquín Carneado	Ruiz	Hospital Universitario Puerta De Hierro, Majadahonda
José González	Ruiz	Hospital De Galdakao-Usansolo, Galdakao
Carlos De Diego	Rus	Hospital Universitario De Torrevieja, Torrevieja
Manuel Anguita	Sánchez	Clínica Cardiologica Dr Anguita, Córdoba
Pere Domènech	Santasusana	Hospital Universitari De Bellvitge, L'Hospitalet de Llobregat
Antonio Javier Trujillo	Santos	Hospital Universitario Santa Lucía, Cartagena
Nuria Basterra	Sola	Complejo Hospitlario De Navarra, Pamplona
Pedro Abizanda	Soler	Hospital Perpetuo Socorro, Albacete
Ruth María Sánchez	Soriano	Hospital Virgen De Los Lirios, Alcoi
Inmaculada Castillo	Valero	Hospital De Sagunto, Sagunto
Almudena	Valle	Hospital Costa Del Sol, Marbella
Emengol	Vallese	Hospital Del Mar, Barcelona
Tomás Ripoll	Vera	Hospital Son Llàtzer, Palma de Mallorca
Miguel Ahumada	Vidal	Hospital General Universitario De Elche, Elche
Switzerland		
Flavio	Acquistapace	Studio Medico Sanacare, Lugano
Peter	Ammann	Kantonsspital St Gallen, St Gallen
Angelo	Auricchio	Cardio Centro Ticino, Lugano
Juerg H	Beer	Kantonsspital Baden AG, Baden
Robert	Bonvini	Clinique des Grangettes, Chene-Bougeries
Alexander	Breitenstein	Universitaetsspital Zuerich, Zuerich
Pascal	Chatelain	Pascal Chatelain Private Practice, Geneva
Giuseppe	Cocco	Practice of Dr med Giuseppe Cocco, Rheinfelden
Stephane	Cook	Hôpital Fribourgeois - Freiburger Spital, Fribourg
Robert	Escher	Regionalspital Emmental AG, Burgdorf
Thomas	Fischer	Kantonsspital Winterthur, Winterthur
Jean-Jacques	Goy	Hôpital Fribourgeois - Freiburger Spital, Fribourg
Jens	Hellermann	Facharzt FMH fuer Innere Medizin und Kardiologie, Schiers
Alexander	Imhof	Spital Region Oberaargau, Langenthal
Stefan	Kradolfer	Facharzt FMH ALLG Medizin, Basel
Mehdi	Namdar	Geneva University Hospital, Geneva

Daniel	Nobel	Spital Wil, Wil
Joerg	Nossen	Luzerner Kantonsspital, Sursee
Cyril	Pellaton	Hôpital neuchâtelois, Neuchatel
Ralf	Polikar	Hôpital de Nyon, Nyon
Jean-Luc	Reny	Hopital Universitaire Geneve, Thônex-Genève
Christoph	Schalcher	Gemeinschaftspraxis Greifensee, Greifensee
Juan	Sztajzel	Clinique de Carouge, Geneva
United Kingdom		
Javed	Ahmed	Freeman Hospital, Newcastle upon Tyne
Ameet	Bakhai	Barnet Hospital, Barnet
Lawrence	Barnes	Rame Medical Limited, Torpoint
Craig	Barr	Russells Hall Hospital, Dudley
Fiona	Bellas	Lambert Medical Centre, Thirsk
Arnold	Berger	Fishermead Medical Centre, Milton Keynes
Daniel	Berkeley	Maryport health services, Maryport
Mark	Bowman	The Ulster Hospital, Belfast
Louise	Buchanan	North Cumbria University Hospitals NHS Trust, Carlisle
Michael	Butler	Waterloo Medical Centre, Blackpool
Philip	Campbell	Royal Gwent Hospital, Newport
Simon	Cartwright	The White Horse Medical Practice, Faringdon
Anoop	Chauhan	Blackpool Victoria Hospital, Blackpool
Anna Maria	Choy	Ninewells Hospital, Dundee
Piers	Clifford	Buckinghamshire Healthcare NHS Trust, High Wycombe
Damien	Cullington	Leeds General Infirmary, Leeds
Mark	Davies	West Cross Medical Centre, Swansea
Anand	Dixit	Freeman Hospital, Newcastle upon Tyne
Savio	D'Souza	Darent Valley Hospital, Dartford
Patrick	Eavis	Heart of Bath Medical Partnership, Bath
Matthew	Fay	Westcliffe Medical Practice, Bradford
William	Francis	Stokesley Health Centre, Stokesley
Ahmet	Fuat	Carmel Surgery, Darlington
Scot	Garg	Royal Blackburn Hospital, Blackburn
Jason	Glover	Basingstoke and North Hampshire Hospital, Basingstoke
Chris	Gregory	Royal Albert Edward Infirmary, Wigan
Gunaratnam	Gunathilagan	Queen Elizabeth the Queen Mother Hospital, Margate
Dhiraj	Gupta	Liverpool Heart and Chest Hospital, Liverpool
David	Hargroves	William Harvey Hospital, Ashford
Simon	Hutchinson	Ballygomartin Group Practice, Belfast
Sachin	Jadhav	Nottingham University Hospitals City Campus, Nottingham
Martin	James	Royal Devon & Exeter Hospital, Exeter
David	Jones	Harefield Hospital, Harefield
Honer	Kadr	Queen's Hospital, Romford
Charalampos	Kartsios	Birmingham Heartlands Hospital, Birmingham
Ravish	Katira	St Helens & Knowsley Teaching Hospital NHS Trust, Prescot
Philip	Keeling	Torbay Hospital, Torquay
Masood	Khan	Watford General Hospital, Watford

Neville	Kukreja	Lister Hospital, Stevenage
Steven	Lindsay	Bradford Royal Infirmary, Bradford
Nicholas	Linker	James Cook University Hospital, Middlesbrough
Graeme	Little	Sleights and Sandsend Medical Practice, Whitby
David	MacDougall	Hairmyres Hospital, East Kilbride
Mehrdad	Malekianpour	University Hospital Wishaw, Wishaw
Aravindakshan	Manoj	Broadgreen Hospital, Liverpool
Paula	Marrett	The Fowey River Practice, Fowey
Sarah	Maxwell	The Crouch Oak Family Practice, Weybridge
Conor	McCann	Belfast City Hospital, Belfast
Sam	McClure	Sunderland Royal Hospital, Sunderland
Terry	McCormack	Whitby Group Practice, Whitby
Kneale	Metcalf	Norfolk and Norwich Hospital, Norwich
Andrew	Moriarty	Craigavon Area Hospital, Co Armagh
Timothy	Myhill	Rothwell & Desborough Healthcare Group, Wellingborough
David	Neilson	Dumbarton Health Centre, Dumbarton
Paul	Nixon	Swan Lane Medical Centre, Bolton
Washik	Parkar	Gloucester House Medical centre, Urmston
Jeremy	Platt	The Binfield Surgery, Binfield
Joanne	Porter	Peterborough City Hopsital, Peterborough
Neeraj	Prasad	Wye Valley NHS Trust-County Hospital, Hereford
Mark	Richardson	Lindum Medical Practice, Lincoln
Duncan	Rogers	Mowbray House Surgery, Northallerton
John	Ryan	The Alverton Practice, Penzance
Manish	Saxena	Barts Health, London
Matthew	Seager	Dr C J C Johns & Partners, Killay
Michael	Seddon	Musgrove Park Hospital, Taunton
Roxy	Senior	Northwick Park Hospital, Harrow
Gabrielle	Slade	Forest End Surgery, Waterlooville
Amrit	Takhar	Wansford and Kings cliffe practice, Peterborough
Swaminathan	Thiagarajan	Pickering Medical Practice, Pickering
Roland	Veltkamp	IMP COLL HEALTH NHST 1, London
Azhar	Zafar	Danes Camp Medical Practice, Northampton
Azfar	Zaman	Freeman Hospital, Newcastle upon Tyne
